# Antimicrobial potential of selected medicinal plants against drug-resistant pathogens: a systematic review

**DOI:** 10.3389/fphar.2026.1735625

**Published:** 2026-07-09

**Authors:** Shuroug A. Alowais, Ruya Alshkrh, Ahmad Abdulaziz Al-Owais, Sahar S. Alghamdi

**Affiliations:** 1 King Saud Bin Abdulaziz University for Health Sciences (KSAU-HS), Riyadh, Saudi Arabia; 2 King Abdullah International Medical Research Center (KAIMRC), Riyadh, Saudi Arabia; 3 Ministry of National Guard Health Affairs (MNGHA), Riyadh, Saudi Arabia; 4 King Saud University (KSU), Riyadh, Saudi Arabia

**Keywords:** antimicrobial activity, antimicrobial resistance, plants, systematic review, traditional medicine

## Abstract

**Background:**

Antimicrobial resistance (AMR) poses a critical global health threat, necessitating the discovery of novel antimicrobial agents. Traditional medicinal plants have long been used for managing infectious diseases, yet scientific validation of their efficacy remains limited for many species. This systematic review synthesizes existing evidence on the antimicrobial activities of nine medicinal plants traditionally used: *Lepidium sativum*, *Saussurea costus, Rhus tripartita*, *Chenopodium murale, Pyrus communis*, *Argemone ochroleuca*, *Trigonella hamosa*, *Galium odoratum*, and *Erucaria hispanica*.

**Methods:**

Following PRISMA guidelines, a comprehensive search was conducted in Google Scholar for studies published between 1 January 2000–1 August 2025. Search terms combined each plant’s scientific name with antimicrobial-related keywords (“antimicrobial,” “antibacterial,” “antifungal”) and study type filters (“*in vitro*,” “*in vivo*”). Only English-language studies investigating the antibacterial or antifungal activity of extracts or isolated metabolites from the target species were included. Clinical trials, reviews, case reports, and non-English publications were excluded. Data extraction captured microorganism type and strain, plant part studied, extraction method, antimicrobial assay, and reported activity (MIC, MBC, or inhibition zone).

**Results:**

A total of fifty-six eligible studies were included in the review. The evidence indicated that antibacterial and antifungal activities varied among the studied species. *L. sativum*, *S. costus*, *R. tripartita*, and *C. murale* were relatively well investigated, with findings showing broad-spectrum activity against both Gram-positive and Gram-negative bacteria. In contrast, *P. communis*, *A. ochroleuca*, and *T. hamosa* demonstrated antibacterial effects on both bacterial groups; however, they require further research. Several plants displayed notable antifungal effects, particularly against *Candida* spp., though results varied depending on extraction method, plant part, and microbial strain. Notably, studies employing advanced extraction techniques such as supercritical fluid extraction and green synthesis of nanoparticles, particularly silver nanoparticles, frequently reported enhanced antimicrobial efficacy compared to conventional solvent extracts. These nanoparticle formulations often exhibited larger inhibition zones and lower MIC values, highlighting their potential to potentiate the bioactivity of plant extracts.

**Conclusion:**

This review highlights the antimicrobial potential of the selected medicinal plants and supports their traditional use in managing infectious diseases. Standardized methodologies and bioactive compound isolation are recommended to facilitate their future development as candidates for combating AMR pathogens.

**Systematic Review Registration:**

https://www.crd.york.ac.uk/PROSPERO/view/CRD420251047619.

## Graphical Abstract

**Figure:**
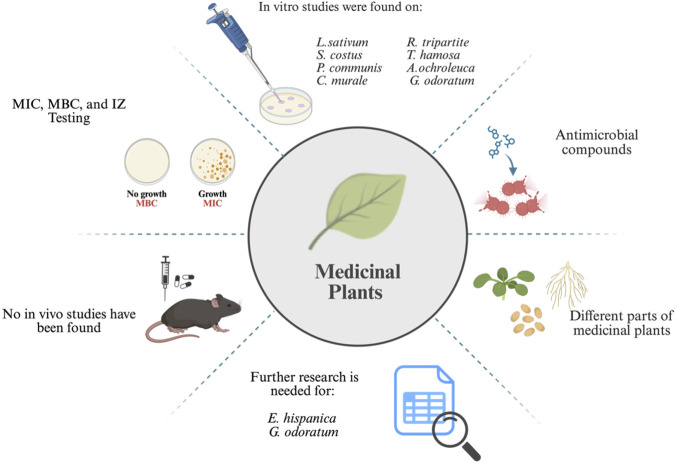

## 1 Introduction

Antimicrobial resistance (AMR) is defined as the ability of pathogenic microbes, such as bacteria, fungi, viruses, and parasites, to resist the effects of antimicrobial agents, rendering these treatments less effective or ineffective. This resistance prevents successful management of infection and worsens clinical outcomes. Globally, the rate of AMR is rising, representing a major global concern. It is estimated that approximately 1.27 million deaths were directly attributable to AMR in 2019 (Murray et al., 2022). Furthermore, between 2018 and 2023, more than 40% of monitored pathogen–antibiotic combinations exhibited increasing antibiotic resistance, with average annual increases ranging from 5% to 15% (Organization, 2025).

AMR is represented by several mechanisms, including the capability of the microbe to generate an enzyme that degrades antimicrobial agents, alterations in metabolic pathways, and modifications to the drug binding sites (Abushaheen et al., 2020). These challenges have driven scientists around the world to seek novel solutions to combat AMR.

Medicinal plants provide a promising field for new therapeutic discoveries, inspired by the traditional uses in treating infectious diseases (Mahady, 2005). *Lepidium sativum*, *Saussurea costus, Rhus tripartita*, *Chenopodium murale, Pyrus communis*, *Argemone ochroleuca*, *Trigonella hamosa*, *Galium odoratum*, and *Erucaria hispanica*, are medicinal plants that have been utilized traditionally for managing infections. Recent studies have identified various secondary metabolites in these plants, such as polyphenols, flavonoids, and steroids, which suggests their potential as antimicrobial agents (Abdel-Aziz et al., 2014; Abdelwahab et al., 2021; Ahmed et al., 2018; Chatoui et al., 2016; Rao et al., 2023; Sharma et al., 2015; Tlili et al., 2021).

The species included in this review were selected because they are traditionally used in various medicinal applications and have demonstrated antimicrobial activity in individual *in vitro* and *in vivo* studies. However, the available evidence remains dispersed across the literature and, to our knowledge, has not been systematically synthesized. Therefore, a focused systematic review was conducted to evaluate and summarize the current evidence regarding their antimicrobial potential.

This systematic review aims to summarize the findings on the selected medicinal plants by screening the current evidence from existing *in vitro* and *in vivo* studies that investigate their antimicrobial activity, with the goal of identifying promising candidates for future drug development against AMR pathogens.

## 2 Methodology

### 2.1 Research question

This systematic review was conducted in accordance with the Preferred Reporting Items for Systematic Reviews and Meta-Analyses (PRISMA) guidelines. The review protocol was registered in the International Prospective Register of Systematic Reviews (PROSPERO; registration number CRD420251047619). The objective of this review was to systematically identify, summarize, and critically evaluate existing *in vitro* and *in vivo* evidence on the antimicrobial potential of selected medicinal plants. To ensure up-to-date and relevant findings, only studies published within the past 25 years (2000–2025) were included.

### 2.2 Data sources and search strategy

A systematic literature search was conducted using Google Scholar as the primary database to select the studies that examine the antimicrobial biological activity of the selected medicinal plants. To conduct the search in the database, the following search strategy was applied for each plant.

“*Saussurea costus*” AND (“antimicrobial” OR “antibacterial” OR “antifungal”) AND (“*in vitro*” OR “*in vivo*”). “*Lepidium sativum*” AND (“antimicrobial” OR “antibacterial” OR “antifungal”) AND (“*in vitro*” OR “*in vivo*”). “*Rhus tripartita*” AND (“antimicrobial” OR “antibacterial” OR “antifungal”) AND (“*in vitro*” OR “*in vivo*”). “*Pyrus communis*” AND (“antimicrobial” OR “antibacterial” OR “antifungal”) AND (“*in vitro*” OR “*in vivo*”). “*Chenopodium murale*” AND (“antimicrobial” OR “antibacterial” OR “antifungal”) AND (“*in vitro*” OR “*in vivo*”). “*Erucaria hispanica*” AND (“antimicrobial” OR “antibacterial” OR “antifungal”) AND (“*in vitro*” OR “*in vivo*”). “*Trigonella hamosa*” AND (“antimicrobial” OR “antibacterial” OR “antifungal”) AND (“*in vitro*” OR “*in vivo*”). “*Argemone ochroleuca*” AND (“antimicrobial” OR “antibacterial” OR “antifungal”) AND (“*in vitro*” OR “*in vivo*”). “*Galium odoratum*” AND (“antimicrobial” OR “antibacterial” OR “antifungal”) AND (“*in vitro*” OR “*in vivo*”). The searche was limited to English-language publications within the selected period 01/01/2000 to 01/08/2024 in English.

### 2.3 Inclusion criteria

Studies were included if they were conducted *in vivo* or *in vitro* and investigated the antibacterial and/or antifungal activity of extracts of the predefined medicinal plants or their isolated metabolites.

### 2.4 Exclusion criteria1

Studies were excluded if they investigated plant species other than the nine predefined plants, did not investigate antimicrobial activity, with study design such as clinical trials, reviews, or case reports, or were published in a language other than English.

### 2.5 Study selection

**FIGURE 1:**
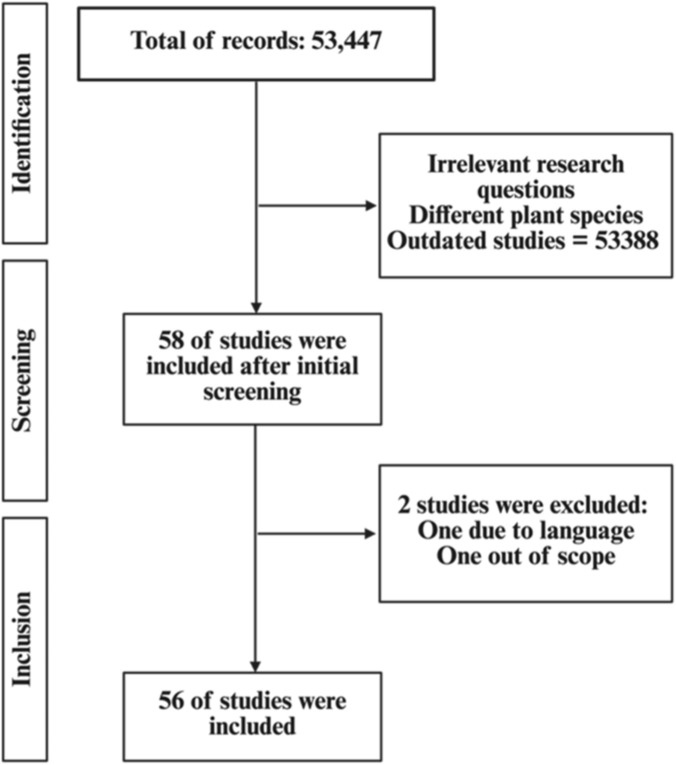
Study selection process.

All records identified through the search strategy were exported and screened for eligibility. Titles and abstracts were initially screened against the predefined inclusion and exclusion criteria. Studies that appeared relevant underwent full-text review. Duplicate records and studies that did not meet the eligibility criteria were excluded. The final selection of studies was independently reviewed by two reviewers, and any disagreements were resolved through discussion and consensus. The study selection process is summarized in the PRISMA flow diagram (Figure 1).

### 2.6 Data extraction

Data extraction was conducted by one reviewer and independently checked by a second reviewer for accuracy. The extracted data included several aspects that provide a clear conclusion regarding the antimicrobial activity of the plant extracts: Microbe name and strain, source of isolates, plant part investigated, extraction method, Minimum Inhibitory Concentration (MIC), Minimum Bactericidal Concentration (MBC), and zone of inhibition.

### 2.7 Taxonomical validation

The plant species included in this study belong to the Kingdom *Plantae*, Phylum *Streptophyta*, Clas *Equisetopsida*, and Subclass *Magnoliidae;* however, they are distributed across several taxonomic orders and families. The taxonomic identity of the selected species was validated using the Plants of the World Online (POWO) database (Table 1). Synonyms for the plant species are provided in the Supplementary Materials (Supplementary Table 1S).

**TABLE 1:** Taxonomic classification of selected plant species included in the study.

Order	Family	Genus	Species
Brassicales	*Brassicaceae*	*Lepidium*	*Lepedium sativum*
Asterales	*Asteraceae*	*Dolomiaea*	*Dolomiaea* costus
Sapindales	*Anacardiaceae*	*Searsia*	*Searsia tripartita*
Caryophyllales	*Amaranthaceae*	*Chenopodiastrum*	*Chenopodiastrum murale*
Rosales	*Rosaceae*	*Pyrus*	*Pyrus communis*
Ranunculales	*Papaveraceae*	*Argemone*	*Argemone ochroleuca*
Fabales	*Fabaceae*	*Trigonella*	*Trigonella glabra*
Gentianales	*Rubiaceae*	*Galium*	*Galium odoratum*
Brassicales	*Brassicaceae*	*Erucaria*	*Erucaria hispanica*

## 3 Results

A total of 53,447 records were identified. Studies that were out of scope, invloved different plant species, or were outdated studies were excluded. Following screening, 58 studies were assessed for eligibility; two were excluded due to language and lack of relavance to the research question, resulting in 56 studies included in the final analysis (Figure 1). The antibacterial and antifungal bioactivity of the selected medicinal plants was evaluated through a systematic review of *in vitro* and *in vivo* studies (Table 2).

**TABLE 2:** Overview of the current studies on the selected medicinal plants.

Plant name	Number of studies identified	Types of experimental approach
*L. sativum*	Twenty studies	*In vitro*
*S. costus*	Thirteen studies	*In vitro*
*R. tripartite*	Six studies	*In vitro*
*C. murale*	Six studies	*In vitro*
*P. communis*	Five studies	*In vitro*
*A. ochroleuca*	Three studies	*In vitro*
*T. hamosa*	Two studies	*In vitro*
*G. odoratum’s*	One study	*In vitro*
*E. hispanica*	No study	Not applicable*

^*^ Not applicable: there is no pre-clinical studies (*in vitro* or *in vivo*) on this plant.

**FIGURE 2:**
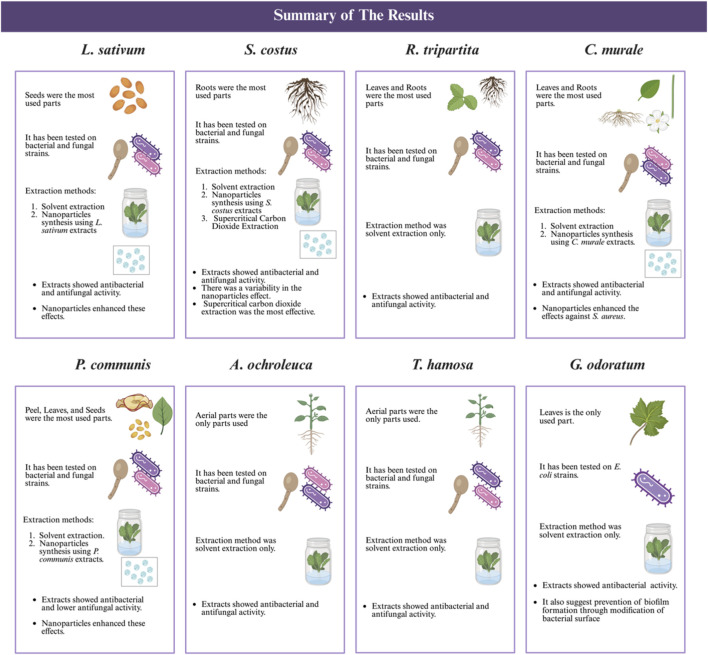
Summary of existing in vitro study findings on the selected plants: most commonly used plant parts, extraction methods, types of pathogenic microbes tested for antimicrobial activity, and observed effects.

A total of 56 *in vitro* studies investigating *Lepidium sativum*, *S. costus, R. tripartita*, *C. murale, P. communis*, *A. ochroleuca*, *Trigonella hamosa*, *G. odoratum*, and *E. hispanica* were identified. These studies evaluated antimicrobial activity against a wide range of bacterial and fungal strains derived from various sources, including standard laboratory strains and clinical isolates (Figure 2).

### 3.1 Lipidium sativum

*Lepidium sativum* is the most extensively investigated plant, with a total of 20 studies evaluating its antibacterial and antifungal activities (Haj Bloukh et al., 2021; El-Sherbiny et al., 2023; Ghebremariam et al., 2018). These investigations assessed its efficacy against Gram-positive and Gram-negative bacteria, as well as various fungal strains. The majority of studies utilized the seeds of *L. sativum*. Extraction was predominantly performed using polar solvents such as methanol, ethanol, n-butanol, and water (Ait-yahia et al., 2018; Al-Shimaa Saber Abd-elmegeed et al., 2023; Zia-Ul-Haq et al., 2011; El-Sherbiny et al., 2023; El-Haggar et al., 2021; Abebe and Mekonnen, 1970). A smaller number of studies employed nonpolar solvents, including ethyl acetate, n-hexane, and chloroform (Aragaw and Nega, 2017; Ghebremariam et al., 2018; Adera et al., 2022). In addition to conventional solvent-based extraction, several studies incorporated alternative methods aimed at enhancing the antimicrobial efficacy *of L. sativum* extracts (Haj Bloukh et al., 2021; Alnehia et al., 2023; Alotaibi et al., 2021).

The studies encompassed a broad range of Gram-positive bacterial strains, including *S. aureus*, *Bacillus subtilis*, *Listeria monocytogenes*, *Streptococcus pneumoniae*, *Streptococcus pyogenes*, *E. faecalis*, *Streptococcus agalactiae*, *Bacillus cereus*, *M. luteus*, *M. roseus*, and *Sarcina lutea*. Among these, *S. aureus* was the most frequently employed organism in assessing the antibacterial activity of *L. sativum*. Solvent-based extracts demonstrated notable antibacterial effects, though with considerable variability in potency. For instance, Ait-Yahia et al. (2018) reported MIC values ranging from 3.5 to 5 mg/mL (Ait-yahia et al., 2018). Other studies assessed antibacterial activity using inhibition zone diameters, categorized as low (6 mm), moderate (10–14 mm), and high (up to 21 mm), as reported by Aragaw and Nega (2017), Ghebremariam et al. (2018), Al-Shimaa Saber Abd-elmegeed et al. (2023), Sayed (2014), Al-Asbahi and Alasbahi (2016), El-Sherbiny et al. (2023), El-Haggar et al. (2021), Abebe and Mekonnen (1970), Adera et al. (2022), and Ibrahim and Kebede (2020).

Alternative extraction and formulation approaches have been also explored to enhance antimicrobial efficacy. For example, Haj Bloukh et al. (2021) synthesized silver nanoparticles using *L. sativum* aqueous extracts and reported enhanced antibacterial activity compared to the crude extract, with inhibition zones increasing from 14 to 20 mm (Haj Bloukh et al., 2021). Similarly, Alnehia et al. (2023) utilized various nanoparticles-based formulations, achieving an inhibition zone of 23 mm. This enhancement pattern was consistent across multiple bacterial strains (Alnehia et al., 2023). In contrast, Rezig et al. (2022), who investigated the antibacterial activity of *L. sativum* oil, reported no observable inhibitory effects (Rezig et al., 2022). The results are summarized in Table 3.

**TABLE 3:** Comprehensive analysis of *L. sativum* reported *in vitro* studies on gram-positive bacteria: treatment protocols and observed effects.

Microbe name	Sources	Part used	Extraction methods	Units	References	Commercial availability
Gram-positive						
*Staphylococcus aureus*	ATCC 25923	Seeds	Aqueous extraction + AgNPs	Inhibition zones Extracts + AgNP (10%, 1.08 μg/mL): 19–20 mm (pH 7 to pH 8.5) Extract (2 mL) at concentrations of 0.0025–0.01 g/mL:13 −14 mm Extracts (0.01 g/mL, agar well method) showed no inhibition (0 mm)	Haj Bloukh et al. (2021)	Not available
	ATCC 25923, ATCC 43300		Methanol, n-Butanol by maceration	IC_50_: 0.47 mg/mL MIC: 4 mg/mL	Ait-yahia et al. (2018)	
	ATCC25923, clinical isolate		Ethanol, chloroform and Methanol by soxhlet extractor	Inhibition zones 19.64–24.40 mm	Aragaw and Nega (2017)	
	ND		Aqueous extraction by maceration + ZnO NP	Inhibition zones at concentrations 60–120 mg/mL 21–23 mm	Alnehia et al. (2023)	
	ATCC 25923		Hexane, ethanol, Methanol by soxhlet	Inhibition zones at concentration 250 mg/mL 8.2–19.9 mm	Ghebremariam et al. (2018)	
	ATCC 25923, ATCC 6538		Oil extraction	NA	Rezig et al. (2022)	
	ATCC 25923, MRSA		Ethanol, acetone, Aqueous	Inhibition zones 7.33–8.33 mm MRSA: 8–8.33 mm	Al-Shimaa Saber Abd-elmegeed et al. (2023)	
	ND		Ethanol	MIC: >10 mg/mL	Zia-Ul-Haq et al. (2011)	
	ND		Aqueous, Methanol, Diethyl-ether, n-Butanol, Ethyl acetate and chloroform	Inhibition zones: 5 mm MIC: >200 μg/mL	Sayed (2014)	
	ATCC 29737		Methanol, Water	Inhibition zones: 12–16 mm	Al-Asbahi and Alasbahi (2016)	
	ATCC-25923		Polysaccharides extraction	Inhibition zones at concentrations 0.25–3 mg/mL 10.39–19.65 mm	Alkahtani et al. (2020)	
	ATCC25923		Aqueous and ethanol	Inhibition zones: 15.4 mm MIC: 55 mg/L	El-Sherbiny et al. (2023)	
	ND		Ethanolic extracts sprouts grown under elevated CO_2_	The heatmap of the results showed increasing of antibacterial activity 0.84–1.17	Alotaibi et al. (2021)	
	ATCC 6538	Seeds + sliver nanoparticles (AgNP)	Ethanol extracts Microwave-Assist Water Extract Ultrasonic-Assist ethanol Extract	Inhibition zones: 8–10 mm	F. Abo El-Maati et al. (2016)	
	ND	Seeds and leaves	Ethanol by maceration	Inhibition zones with different extracts 15.2–21.6 mm MIC: 0.9 mg/mL	El-Haggar et al. (2021)	
	ND	Leaves	Methanol by maceration	Inhibition zones: 6 mm	Abebe and Mekonnen (1970)	
	ND		Petroleum ether by soxhlet	Inhibition zones: 18.50 mm For the leaf extracts MIC and MBC: 0.05 μg/mL For the seeds oil extracts MIC: 0.10–0.20 μg/mL MBC was 0.20 μg/mL	Adera et al. (2022)	
	ATCC25923		Methanol and Aqueous by maceration	Inhibition zones: 20 mm	Ibrahim and Kebede (2020)	
*Bacillus subtilis*	WDCM 0003	Seeds	Aqueous extraction + AgNPs	Extracts + AgNP (10%, 1.08 μg/mL): 18 mm at both pH 7 and pH 8.5 Extracts (2 mL) at concentrations of 0.0025–0.01 g/mL: 10–13 mm Extracts (0.01 g/mL, agar well method) showed no inhibition (0 mm)	Haj Bloukh et al. (2021)	
	ND		Ethanolic extracts sprouts grown under elevated CO_2_	The heatmap of the results showed increasing of antibacterial activity 0.28–0.70	Alotaibi et al. (2021)	
	ND		Ethanol	MIC: >10 mg/mL	Zia-Ul-Haq et al. (2011)	
	ND		Aqueous, Methanol, Diethyl-ether, n-Butanol, Ethyl acetate and chloroform	Inhibition zones: 6 mm MIC: >200 μg/mL	Sayed (2014)	
	ND	Seeds and Leaves	Ethanol by maceration	Inhibition zones: with different extracts 17.2 MIC: 5.2 mg/mL	El-Haggar et al. (2021)	
*Listeria monocytogenes*	ScottA	Seeds + sliver nanoparticles (AgNP)	Ethanol extracts Microwave-Assist Water Extract Ultrasonic-Assist ethanol Extract	Inhibition zones: 9–10 mm	F. Abo El-Maati et al. (2016)	
	ATCC 15313	Seeds	Oil extraction	NA	Rezig et al. (2022)	
	ND	Leaves	Methanol by maceration	Inhibition zones: 6 mm	Abebe and Mekonnen (1970)	
*Streptococcus pneumoniae*	ATCC 49619	Seeds	Aqueous extraction + AgNPs	Extracts + AgNP (10%, 1.08 μg/mL): 14–15 mm (pH 7 to pH 8.5) Extracts (2 mL) at concentrations of 0.0025–0.01 g/mL: 10–12 mm Extracts (0.01 g/mL, agar well method): 12 mm	Haj Bloukh et al. (2021)	
	ATCC63 and clinical isolate		Ethanol, chloroform and Methanol by soxhlet extractor	Inhibition zones 24.17–27.38 mm	Aragaw and Nega (2017)	
*Proteus mirabilis*	ND	Seeds	Ethanol	MIC: >1 mg/mL	Zia-Ul-Haq et al. (2011)	
	ATCC 29906		Aqueous extraction + AgNPs	Extracts + AgNP (10%, 1.08 μg/mL): 10 mm at both pH 7 and pH 8.5 Extracts (2 mL) at concentrations of 0.0025–0.01 g/mL (0 mm) Extracts (0.01 g/mL, agar well method) (0 mm)	Haj Bloukh et al. (2021)	
*Streptococcus pyogenes*	ATCC 19615	Seeds	Aqueous extraction + AgNPs	Extracts + AgNP (10%, 1.08 μg/mL): 14–15 mm (pH 7 to pH 8.5) Extracts (2 mL) at concentrations of 0.0025–0.01 g/mL: 9–11 mm Extracts (0.01 g/mL, agar well method): 10 mm	Haj Bloukh et al. (2021)	
*Enterococcus faecalis*	ATCC 29212	Seeds	Aqueous extraction + AgNPs	Inhibition zones ranged from 0 mm (2 mL of 0.0025–0.01 g/mL) increased to 12 mm using Extracts (0.01 g/mL, agar well method) further increased to 14–15 mm with 10% Extracts + AgNP (1.08 μg/mL) at pH 7–8.5	Haj Bloukh et al. (2021)	
			Methanol, n-Butanol by maceration	IC_50_:0.4 mg/mL MIC: 3.5 mg/mL	Ait-yahia et al. (2018)	
*Bacillus cerreus*	ATCC 11778	Seeds	Oil extraction	NA	Rezig et al. (2022)	
*Micrococcus luteus*	ND	Seeds	Ethanol	MIC: >10 mg/mL	Zia-Ul-Haq et al. (2011)	
*Micrococcus roseus*	ND	Seeds	Aqueous, Methanol, Diethyl-ether, n-Butanol, Ethyl acetate and chloroform	Inhibition zones: 8 mm MIC: >200 μg/mL	Sayed (2014)	
*Sarcina lutea*	ND	Seeds	Ethanolic extracts sprouts grown under elevated CO_2_	The heatmap of the results showed increasing of antibacterial activity 0.08–0.75	Alotaibi et al. (2021)	
*Streptococcus agalactiae*	ATCC 12338	Leaves	Methanol and Aqueous by maceration	Inhibition zones: 10–15 mm	Ibrahim and Kebede (2020)	

ND (Not determined): The studies did not report the source of pathogenic microbe.

NA (No activity): The studies report that the extracts have no activity against the tested microbes.

A variety of Gram-negative bacterial strains were included in studies evaluating the antibacterial potential of *L. sativum*, such as *Escherichia coli*, *P. aeruginosa*, *K. pneumoniae*, *Salmonella typhi*, *Brucella abortus*, *S. Enteritidis*, *Serratia marcescens*, *Shigella dysenteriae*, *S. enterica*, *E. faecium*, *S. Typhimurium*, *Proteus mirabilis*, *Shigella flexneri*, and *S. boydii*. Among these, *P. aeruginosa*, *E. coli*, and *K. pneumoniae* were the most frequently employed strains for assessing antibacterial efficacy. Similar to Gram-positive bacteria, extracts of *L. sativum* exhibited variable antibacterial activity against Gram-negative strains depending on the extraction solvents used. Reported inhibition zones ranged from 6 to 24 mm, as reported by Alnehia et al. (2023), Ghebremariam et al. (2018), El-Haggar et al. (2021), and Adera et al. (2022). Notably, the incorporation of nanoparticle synthesis significantly enhanced the antibacterial effects. However, *P. mirabilis* exhibited resistance even to nanoparticle-based formulations (Table 4) (Haj Bloukh et al., 2021).

**TABLE 4:** Comprehensive analysis of *Lepidium sativum* reported *in vitro* studies on gram-negative bacteria: treatment protocols and observed effects.

Microbe name	Sources	Part used	Extraction methods	Units	References	Commercial availability
Gram-negative						
*Escherichia coli*	WDCM 00013 Vitroids	Seeds	Aqueous extraction + AgNPs	Extracts + AgNP (10%, 1.08 μg/mL): 17 mm at both pH 7 and pH 8.5 Extracts (2 mL) at concentrations of 0.0025–0.01 g/mL:13–15 mm Extracts (0.01 g/mL, agar well method): (0 mm)	Haj Bloukh et al. (2021)	Not available
	ATCC 25992		Methanol, n-Butanol by maceration	IC_50_: 0.41 mg/mL MIC: 5 mg/mL	Ait-yahia et al. (2018)	
	ATCC2592, clinical isolate		Ethanol, chloroform and Methanol by soxhlet extractor	Inhibition zones: 11.20 9.60–11.20 mm	Aragaw and Nega (2017)	
	ND		Aqueous extraction by maceration + ZnO NP	Inhibition zones at concentrations 60–120 mg/mL 21–23 mm	Alnehia et al. (2023)	
	ATCC 25922		Oil extraction	NA	Rezig et al. (2022)	
	ATCC 25922, ESβL		Ethanol, acetone Aqueous	Inhibition zones: 7–15.50 mm EsβL: 7.17–8.17 mm	Al-Shimaa Saber Abd-elmegeed et al. (2023)	
	ATCC 25922		Hexane, ethanol, Methanol by soxhlet	Zone inhibition at concentration (250 mg/mL) 8–24 mm, n-hexane were not effective	Ghebremariam et al. (2018)	
	ND		Ethanol	MIC: >15 mg/mL	Zia-Ul-Haq et al. (2011)	
	ND		Aqueous, Methanol, Diethyl-ether, n-Butanol, Ethyl acetate and chloroform	Inhibition zones: 11 mm MIC: >200 μg/mL	Sayed (2014)	
	ATCC-25992		Polysaccharides extraction	Inhibition zones at concentration 0.25–3 mg/mL 6.89–13.91 mm	Alkahtani et al. (2020)	
	ND		Ethanolic extracts sprouts grown under elevated CO_2_	The heatmap of the results showed increasing of antibacterial activity 0.16–61	Alotaibi et al. (2021)	
	ATCC 8739	Seeds + sliver nanoparticles (AgNP	Ethanol extracts Microwave-Assist Water Extract Ultrasonic-Assist ethanol Extract	Inhibition zones: 9–12 mm	F. Abo El-Maati et al. (2016)	
	ND	Seeds and leaves	Ethanol by maceration	Inhibition zones with different extracts 14.5–20.3 mm MIC: 0.85–18.3 mg/mL	El-Haggar et al. (2021)	
	ND		Petroleum ether by soxhlet	For the leaf oil extracts MIC and MBC: 0.20–0.40 μg/mL For the seeds oil extracts MIC and MBC 0.75–1.25 μg/mL	Adera et al. (2022)	
	ND	Leaves	Methanol by maceration	Inhibition zones: 7 mm	Abebe and Mekonnen (1970)	
*Pseudomonas aeruginosa*	WDCM 00026 Vitroids	Seeds	Aqueous extraction + AgNPs	Extracts + AgNP (10%, 1.08 μg/mL): 22 mm at both pH 7 and pH 8.5 Extracts (2 mL) at concentrations of 0.0025–0.01 g/mL: 15–17 mm Extracts (0.01 g/mL, agar well method): (0 mm)	Haj Bloukh et al. (2021)	
	ATCC 27852		Methanol, n-Butanol by maceration	IC_50_: 0.42 mg/mL MIC: 4 mg/mL	Ait-yahia et al. (2018)	
	ATCC 27853		Oil extraction	NA	Rezig et al. (2022)	
	ND		Ethanolic extracts sprouts grown under elevated CO_2_	The heatmap of the results showed increasing of antibacterial activity 0.24–0.75	Alotaibi et al. (2021)	
	NCTC 10662, MβL		Ethanol, acetone Aqueous	Inhibition zones: 7–9.17 mm MβL: 7–7.17 mm	Al-Shimaa Saber Abd-elmegeed et al. (2023)	
	ND		Ethanol	MIC: >10 mg/mL	Zia-Ul-Haq et al. (2011)	
	ND		Methanol, Diethyl-ether, Ethyl acetate and chloroform	Inhibition zones: 8 mm MIC: >200 μg/mL	Sayed (2014)	
*Klebsiella pneumoniae*	WDCM 00097 Vitroids	Seeds	Aqueous extraction + AgNPs	Extracts + AgNP (10%, 1.08 μg/mL): 20 mm at both pH 7 and pH 8.5 Extracts (2 mL) at concentrations of 0.0025–0.01 g/mL: 12–14 mm Extracts (0.01 g/mL, agar well method): (0 mm)	Haj Bloukh et al. (2021)	
	ATCC13883		Ethanol, chloroform and Methanol by soxhlet extractor	Inhibition zones: 19.15–20.75 mm	Aragaw and Nega (2017)	
	ATCC 35657		Oil extraction	NA	Rezig et al. (2022)	
	ND		Methanol, Diethyl-ether, Ethyl acetate and chloroform	Inhibition zones: 8 mm MIC: >200 μg/mL	Sayed (2014)	
	ND	Seeds and leaves	Ethanol by maceration	Inhibition zones with different extracts 15.3–25.3 mm MIC: 8.1–9.7 mg/mL	El-Haggar et al. (2021)	
*Salmonella typhi*	ND	Seeds	Ethanol	MIC: >10 mg/mL	Zia-Ul-Haq et al. (2011)	
	ATCC13311	Leaves	Methanol and Aqueous by maceration	Inhibition zones: 10–25 mm	Ibrahim and Kebede (2020)	
*Shigella dysenteriae*	ND	Seeds	Methanol, Diethyl-ether, Ethyl acetate and chloroform	Inhibition zones: 8 mm MIC: >200 μg/mL	Sayed (2014)	
*Enterococcus faecium*	19,434	Seeds	Oil extraction	NA	Rezig et al. (2022)	
*Salmonella Typhimurium*	ATCC23852	Seeds	Aqueous and ethanol	Zone of inhibition: 16.4 mm MIC: 55 mg/L	El-Sherbiny et al. (2023)	
*Proteus mirabilis*	ATCC 29906	Seeds	Aqueous extraction + AgNPs	NA	Haj Bloukh et al. (2021)	
*Shigella flexneri*	ATCC12022	Seeds	Ethanol, chloroform and Methanol by soxhlet extractor	Inhibition zones: 13.52–18.72 mm	Aragaw and Nega (2017)	
*Shigella bodyii*	ATCC9202	Leaves	Methanol and Aqueous by maceration	Inhibition zones: 10–20 mm	Ibrahim and Kebede (2020)	
*Brucella abortus*	ND	Seeds	Water, ethanol, Hexane	Inhibition zones: 46 mm	Ali (2020)	
*Salmonella*	ND	Leaves	Methanol by maceration	Inhibition zones: 6 mm	Abebe and Mekonnen (1970)	
*Salmonella Enteritidis*	PT4	Seeds of + sliver nanoparticles (AgNP)	Ethanol extracts Microwave-Assist Water Extract Ultrasonic-Assist ethanol Extract	Inhibition zones: 15–20 mm	F. Abo El-Maati et al. (2016)	
*Serratia marcescens*	ND	Seeds of Lepidium sativum + sliver nanoparticles (AgNP)	Ethanol extracts Microwave-Assist Water Extract Ultrasonic-Assist ethanol Extract	Inhibition zones: 7–9 mm	F. Abo El-Maati et al. (2016)	

ND (Not determined): The studies did not report the source of pathogenic microbe.

NA (No activity): The studies report that the extracts have no activity against the tested microbes.

In addition to bacteria, the antifungal activity of *L. sativum* has also been investigated. The most frequently tested fungal species was *C. albicans* (Rezig et al., 2022; Haj Bloukh et al., 2021; Ghebremariam et al., 2018; Zia-Ul-Haq et al., 2011; Miran et al., 2024; Adera et al., 2022; El-Haggar et al., 2021). Fewer studies explored its effects against other fungi, including *A. niger*, *Fusarium solani*, *M. phaseolina*, *S. cerevisiae*, *Trichophyton rubrum*, *A. flavus*, *A. effusus*, *A. parasiticus*, *Fusarium oxysporum*, and *Yersinia aldovae*. The majority of these studies reported positive antifungal activity of *L. sativum* extracts. For example, seed extracts demonstrated antifungal activity against *C. albicans*, with inhibition zones ranging from 8 to 20 mm at a concentration of 250 mg/mL (Ghebremariam et al., 2018). Similarly, leaf extracts exhibited inhibition zones ranging from 15.2 to 26.3 mm (El-Haggar et al., 2021). However, one study by Rezig et al. (2022) noted the absence of antifungal effects in their tested extracts (Table 5) (Rezig et al., 2022).

**TABLE 5:** Comprehensive analysis of *L. sativum* reported *in vitro* studies on fungi: treatment protocols and observed Effects.

Microbe name	Sources	Part used	Extraction methods	Units	References	Commercial availability
Fungi						
*Candida albicans*	WDCM 00054	Seeds	Aqueous extraction + AgNPs	Extracts + AgNP (10%, 1.08 μg/mL):12–13 mm (pH 7 to pH 8.5)	Haj Bloukh et al. (2021)	Not available
	ATCC 10231		Oil extraction	NA	Rezig et al. (2022)	
	ATCC 10231		Hexane, ethanol, Methanol by soxhlet	Zone inhibition at concentration (250 mg/mL) 8–20 mm, Aqueous were not effective	Ghebremariam et al. (2018)	
	ND		Ethanol	MIC: >10 mg/mL	Zia-Ul-Haq et al. (2011)	
	ATCC 18804		Aqueous + sliver nanoparticles	Inhibition zone: 11–14 mm MIC: 1.75–2.03 ppm	Miran et al. (2024)	
	ND	Leaves	Petroleum ether by soxhlet	For the leaf oil extracts MIC and MBC: 0.40–1.75 μg/mL	Adera et al. (2022)	
	ND	Seeds and Leaves	Ethanol by maceration	Zone Inhibition with different extracts 15.2–26.3 mm MIC: 0.9–8.1 mg/mL	El-Haggar et al. (2021)	
*Aspergillus Niger*	ND	Seeds	Ethanol	MIC: >5 mg/mL	Zia-Ul-Haq et al. (2011)	
	ND	Leaves	Petroleum ether by soxhlet	Inhibition zones: 18.50 mm MIC and MFC 0.10–0.50 μg/mL	Adera et al. (2022)	
*Aspergillus flavus*	ND	Seeds and Leaves	Ethanol by maceration	Inhibition zones with different extracts 8.3–20.3 mm MIC: 3.4–12.5 mg/mL	El-Haggar et al. (2021)	
*Fusarium solani*	ND	Seeds	Ethanol	MIC: >10 mg/mL	Zia-Ul-Haq et al. (2011)	
*Macrophomina phaseolina*	ND	Seeds	Ethanol	MIC: >10 mg/mL	Zia-Ul-Haq et al. (2011)	
*Saccharomyces cerevisiae*	ND	Seeds	Ethanol	MIC: >25 mg/mL	Zia-Ul-Haq et al. (2011)	
*Trichophyton rubrum*	ND	Seeds	Ethanol	MIC: >10 mg/mL	Zia-Ul-Haq et al. (2011)	
*Aspergillus effusus*	ND	Seeds	Ethanol	MIC: >25 mg/mL	Zia-Ul-Haq et al. (2011)	
*Aspergillus parasiticus*	ND	Seeds	Ethanol	MIC: >10 mg/mL	Zia-Ul-Haq et al. (2011)	
*Yersinia aldovae*	ND	Seeds	Ethanol	MIC: >25 mg/mL	Zia-Ul-Haq et al. (2011)	

ND (Not determined): The studies did not report the source of pathogenic microbe.

NA (No activity): The studies report that the extracts have no activity against the tested microbes.

### 3.2 Saussurea costus

The antimicrobial potential of *S. costus* has been investigated in 13 studies, targeting Gram-positive and Gram-negative bacteria, as well as various fungal species (Binobead et al., 2024; Al-Zayadi et al., 2023; Soliman et al., 2022). In most studies, the roots were the primary plant part examined, with the exception of one study that utilized powdered *S. costus*. A variety of extraction techniques were employed, including maceration and ultrasonication, using solvents of differing polarities (Sagar et al., 2017; Deabes et al., 2021; Soliman et al., 2022). Solvent-based extraction was the predominant method, with polar solvents such as methanol, ethanol, *n*-butanol, and water being most commonly used. In contrast, a smaller number of studies employed non-polar solvents, including n-hexane, dichloromethane, and ethyl acetate. Additionally, several studies incorporated innovative extraction strategies, such as nanoparticles synthesis using methanolic extracts of *S. costus*, and the applicatioin of supercritical carbon dioxide extraction for isolating costus oil (Al-brahim, 2023; Amina et al., 2020; Ahmed et al., 2022).

Gram-positive bacterial strains investigated in relation to *S. costus* antimicrobial activity include *S. aureus*, *Bacillus subtilis*, *Bacillus cereus*, *Streptococcus pneumoniae*, *Porphyromonas gingivalis*, *E. faecalis*, *Streptococcus mutans*, *Streptococcus pyogenes*, *S. epidermidis*, and *S. sciuri*. *Staphylococcus aureus* was the most frequently studied organism. Solvent-based extractions of *S. costus* generally demonstrated antibacterial activity, particularly against *S. aureus*, with inhibition zones ranging from 7 to 16.5 mm (Binobead et al., 2024; Mammate et al., 2023; Idriss et al., 2023; Deabes et al., 2021; Al-Zayadi et al., 2023).

In addition to conventional extraction methods, other approaches such as nanoparticle synthesis and supercritical carbon dioxide extraction of costus oil revealed variable results. Methanolic and aqueous extracts combined with iron oxide nanoparticles exhibited comparable activity to the crude extracts, with inhibition zones of 7–9 mm, and no activity was observed against *B. subtilis* (Al-brahim, 2023). Conversely, methanol-based magnesium oxide (MgO) nanoparticles showed no inhibition against *S. aureus*, but exhibited activity against *B. subtilis*, with zones ranging from 9 to 17 mm (Amina et al., 2020). Remarkably, supercritical carbon dioxide extraction of costus oil demonstrated significantly higher antibacterial efficacy, with inhibition zones of 40–52 mm against *S. aureus* and 45–50 mm against *B. subtilis* (Ahmed et al., 2022).

Gram-negative bacterial strains evaluated for their susceptibility to *S. costus* extracts included *Escherichia coli*, *P. aeruginosa*, *K. pneumoniae*, *S. enterica*, *Salmonella typhi*, *Shigella* spp., and *Neisseria* spp. Among these, *E. coli*, *P. aeruginosa*, and *K. pneumoniae* were the most frequently studied organisms. Similar to the results observed with Gram-positive strains, solvent-based extracts of *S. costus* demonstrated antibacterial activity against Gram-negative bacteria, with inhibition zones ranging from 6 to 16.5 mm (Binobead et al., 2024; Al-Zayadi et al., 2023; Idriss et al., 2023; Deabes et al., 2021).

Innovative extraction approaches also revealed varying degrees of antibacterial efficacy. For *E. coli*, methanolic and aqueous extracts combined with iron oxide nanoparticles, as well as methanol-based MgO nanoparticles, exhibited inhibition zones of 8–9 mm (Al-brahim, 2023; Amina et al., 2020). Against *P. aeruginosa*, methanol-based MgO nanoparticles showed no activity, whereas iron oxide nanoparticle formulations displayed a moderate inhibition zone of 11 mm. Supercritical carbon dioxide extraction of costus oil produced notably higher antibacterial effects, with inhibition zones of 32–40 mm against *E. coli*, 28–42 mm against *P. aeruginosa*, and 7–20 mm against *K. pneumoniae* (Ahmed et al., 2022).

A diverse range of fungal species has been evaluated for susceptibility to *S. costus* extracts, including *C. albicans*, *C. tropicalis*, *A. niger*, *A. flavus*, *A. parasiticus*, *A. carbonarius*, *A. ochraceus*, *Fusarium verticillioides*, *Fusarium proliferatum*, *P. verecossum*, *Yersinia* spp., *Alternaria* spp., *Rhizopus* spp., *C. parapsilosis*, *C. pseudotropicalis*, *C. guilliermondii*, and *C. glabrata* (Soliman et al., 2022; Idriss et al., 2023; Ahmed et al., 2022; Sagar et al., 2017). Solvent-based extractions demonstrated substantial antifungal activity. In a study by Soliman et al. (2022), multiple solvents—methanol, n-hexane, n-butanol, dichloromethane, and ethyl acetate—were employed, yielding inhibition zones of 10–15 mm against *C. albicans*, *C. tropicalis*, *C. parapsilosis*, *C. pseudotropicalis*, and *C. guilliermondii* (Soliman et al., 2022). Similarly, Deabes et al. (2021) utilized ethanol via ultrasonication, reporting inhibition zones ranging from 9 to 17 mm against several filamentous fungi, including *A. niger*, *A. flavus*, *A. parasiticus*, *A. carbonarius*, *A. ochraceus*, *F. verticillioides*, *F. proliferatum*, and *P. verecossum* (Deabes et al., 2021).

Nanoparticle-based formulations also demonstrated enhanced antifungal efficacy. Amina et al. (2020) reported inhibition zones of 20 mm and 19 mm against *C. albicans* and *C. glabrata*, respectively (Amina et al., 2020). In contrast, the supercritical carbon dioxide extraction of costus oil showed moderate antifungal effects, producing inhibition zones of 12–15 mm against *C. albicans*, but no observable activity against *A. flavus* and *Fusarium oxysporum* (Ahmed et al., 2022). In another investigation, nanoparticles prepared by Al-Brahim (2023) were only effective against *A. niger* and *Alternaria* spp., while *Rhizopus* spp. Exhibited resistance (Table 6) (Al-brahim, 2023).

**TABLE 6:** Comprehensive analysis of *S. costus* reported *in vitro* studies: treatment protocols and observed effects.

Microbes	Sources	Part used	Extract method	Units	References	Commercial availability
Gram-positive						
*Staphylococcus aureus*	ND	Roots	Methanol, ethanol, acetone	Inhibition zones: 15.30–25.33 mm	Sagar et al. (2017)	Not available
	MTCC-29213		Methanol	Inhibition zones: 11–18 mm MIC: 7.81 μL/mL MBC: 15.6 μL/mL	Binobead et al. (2024)	
	ND		70% ethanol, 100% water	Inhibition zones Aqueous 400 mg/mL (2 mg/disc) 8.01 mm 200 mg/mL (1 mg/disc) 9.02 mm Ethanolic 400 mg/mL (2 mg/disc) 14 mm 200 mg/mL (2 mg/disc) 12.03 mm	Mammate et al. (2023)	
	ND		Methanol and aqueous + Iron oxide nanoparticles	Inhibition zones: 7–9 mm	Al-brahim (2023)	
	ATCC 25923		Methanol MgO nanoparticles	NA	Amina et al. (2020)	
	MRSA ATCC43300		Supercritical Carbon Dioxide Extraction of Costus Oil	Inhibition zones: 40–52 mm	Ahmed et al. (2022)	
	ATCC BAA 1026		Aqueous, acetic acid	Inhibition zones: 16.5 mm MIC and MBC: 25 mg/mL	Idriss et al. (2023)	
	ATCC 13565	Powder	Ethanol ultra-sonicated for 40 min	Inhibition zones at concentration 10–50 μL/mL 16.5–23 mm MIC: 0.2 mg/mL	Deabes et al. (2021)	
*Bacillus subtilis*	MTCC-10400	Roots	Methanol	Inhibition zones: 11–20 mm MIC: 15.6 μL/mL MBC: 31.3 μL/mL	Binobead et al. (2024)	
	ND		Ethanol	Inhibition zones at concentrations 50–100 mg/mL 14–15 mm	Al-Zayadi et al. (2023)	
	ND		Methanol and aqueous + Iron oxide nanoparticles	NA	Al-brahim (2023)	
	ATCC 6633		Methanol MgO nanoparticles	Inhibition zones: 9–17 mm	Amina et al. (2020)	
	ATCC6633		Supercritical Carbon Dioxide Extraction of Costus Oil	Inhibition zones: 45–50 mm	Ahmed et al. (2022)	
*Bacillus cereus*	EMCC 1080	Powder	Ethanol ultra-sonicated for 40 min	Inhibition zones at concentration 10–50 μL/mL 18.5–22.8 mm MIC 0.2 mg/mL	Deabes et al. (2021)	
*Streptococcus penumoniae*	ND	Roots	Ethanol	Inhibition zones at concentrations 50–200 mg/mL 16–18 mm	Azeez Akoul and Ghreeb (2022)	​
*Porphyromonas gingivalis*	ATCC® 53,978	Roots	Aqueous extracts	MIC: 56.2 g/mL	BinShabaib et al. (2022)	
*Enterococcus faecalis*	ATCC® 29,212	Roots	Aqueous extracts	MIC: 56.6 g/mL	BinShabaib et al. (2022)	
*Streptococcus mutans*	ATCC® 55,676	Roots	Aqueous extracts	MIC: 80.5 g/mL	BinShabaib et al. (2022)	
*Streptococcus pyogene*	ND	Roots	Ethanol	Inhibition zones at concentrations: 50–100 mg/mL 16–17 mm	Al-Zayadi et al. (2023)	
*Staphylococcus epidermidis*	MTCC-12228	Roots	Methanol	Inhibition zones: 10–20 mm MIC 10 μL/mL MBC 15.6 μL/mL	Binobead et al. (2024)	
*Staphylococcus sciuri*	ND	Powder	Ethanol ultra-sonicated for 40 min	Inhibition zones at concentrations at 10–50 μL/mL 18.5–21.3 mm MIC 0.2 mg/mL	Deabes et al. (2021)	​
Gram-negative						
*Escherichia coli*	ATCC-25922	Roots	Methanol, ethanol, acetone	Inhibition zones: 14.33–22 mm	Sagar et al. (2017)	Not available
	ATCC-25922		Methanol	Inhibition zones: 6–19 mm MIC 62.5 μL/mL MBC 125 μL/mL	Binobead et al. (2024)	
	ND		Ethanol	Inhibition zones at concentrations: 50–100 mg/mL 17–16 mm	Al-Zayadi et al. (2023)	
	ND		70% ethanol, 100% water	NA	Mammate et al. (2023)	
	ND		Methanol and aqueous + Iron oxide nanoparticles	Inhibition zones: 8–9 mm	Al-brahim (2023)	
	ND		Ethanol	Inhibition zones at concentrations: 50–200 mg/mL 11–13 mm	Azeez Akoul and Ghreeb (2022)	
	ATCC 25966		Methanol MgO nanoparticles	Inhibition zones: 8 mm	Amina et al. (2020)	
	ATCC25922		Supercritical Carbon Dioxide Extraction of Costus Oil	Inhibition zones: 32–40 mm	Ahmed et al. (2022)	
​	ATCC 9637	​	Aqueous, acetic acid	Inhibition zones 14 mm MIC: 25 mg/mL MBC: 50 mg/mL	Idriss et al. (2023)	​
	O157-H7 ATCC 51659	Powder	Ethanol ultra-sonicated for 40 min	Inhibition zones concentrations 10–50 μL/mL: 11.25–15.5 mm MIC 0.3 mg/mL	Deabes et al. (2021)	
*Pseudomonas aeruginosa*	MTCC-27853	Roots	Methanol	Inhibition zones: 7–19 mm MIC 62.5 μL/mL MBC 125 μL/mL	Binobead et al. (2024)	
	ND		Ethanol	Inhibition zones: 50–100 mg/mL 15–14 mm	Al-Zayadi et al. (2023)	
	ND		70% ethanol, 100% water	NA	Mammate et al. (2023)	
	ND		Methanol and aqueous + Iron oxide nanoparticles	Inhibition zones: 8–11 mm	Al-brahim (2023)	
	ATCC 27853		Methanol MgO nanoparticles	NA	Amina et al. (2020)	
	ATCC27853		Supercritical Carbon Dioxide Extraction of Costus Oil	Inhibition zones: 28–42 mm	Amina et al. (2020)	
	ATCC 10145		Aqueous, acetic acid	Inhibition zones: 13.5 mm MIC: 25 mg/mL MBC: 50 mg/mL	Idriss et al. (2023)	
	NRRL B-272	Powder	Ethanol ultra-sonicated for 40 min	Inhibition zones at concentrations: 10–50 μL/mL 14.3–16.3 mm MIC: 0.1 mg/mL	Deabes et al. (2021)	
*Klebsiella pneumoniae*	MTCC-13883	Roots	Methanol	Inhibition zones: 7–20 mm MIC 125 μL/mL MBC 250 μL/mL	Binobead et al. (2024)	
	ND		Ethanol 70%	Inhibition zone Ethanolic 400 mg/mL (2 mg/disc) 12.04 mm 200 mg/mL (2 mg/disc) 12 mm	Mammate et al. (2023)	
			Methanol and aqueous + Iron oxide nanoparticles	NA	Al-brahim (2023)	
	RCMB005 001 (2)		Supercritical Carbon Dioxide Extraction of Costus Oil	Inhibition zones: 49–50 mm	Ahmed et al. (2022)	
*Salmonella entrica*	ATCC 14028	Roots	Aqueous, acetic acid	Inhibition Zones 25 mm MIC: 25 mg/mL MBC: 50 mg/mL	Idriss et al. (2023)	​
	ND	Powder	Ethanol ultra-sonicated for 40 min	Inhibition zones at concentrations 10–50 μL/mL 13.3–17 mm MIC 0.1 mg/mL	Deabes et al. (2021)	
*Salmonella typhi*	ATCC 15566	Powder	Ethanol ultra-sonicated for 40 min	Inhibition zones at concentrations 10–50 μL/mL: 10–13 mm MIC: 0.2 mg/mL	Deabes et al. (2021)	
*5 multidrug resistance isolates* *M. bovis*	5 multidrug resistance isolates	Roots	Methanol	MIC_50_ : 4.513 mg/mL MIC_80_: 7.516 mg/mL	Biswas et al. (2010)	
*Shigella sp*	ND	Roots	Methanol and aqueous + Iron oxide nanoparticles	Inhibition zones: 6 mm	Al-brahim (2023)	
*Neisseria spp*	5 multidrug resistance isolates	Roots	Methanol	MIC50: 12.201 mg/mL MIC_80_: 28.996 mg/mL	Biswas et al. (2010)	
Fungi						
*Candida albicans*	ND	Roots	Methanol n-Hexane, n-Butanol, Dichloromethane, and Ethyl acetate	Inhibition zones: 10–13 mm MIC: 1 mg/mL	Soliman et al. (2022)	Not available
	ATCC 10231	Roots	Aqueous, acetic acid	Inhibition zones: 38 mm MIC: 25 mg/mL MFC: 50 mg/mL	Idriss et al. (2023)	
	ATCC 10231	Roots	Supercritical Carbon Dioxide Extraction of Costus Oil	Inhibition zones: 17–18 mm	Ahmed et al. (2022)	
*Candida tropicalis*	ND	Roots	Methanol n-Hexane, *n*-Butanol, dichloromethane, and ethyl acetate	Inhibition zones: 0.9–12 mm MIC: 1–4 mg/mL	Soliman et al. (2022)	
	RCMB005 004		Methanol MgO nanoparticles	Inhibition zones: 20 mm	Amina et al. (2020)	
	ATCC 10231		Supercritical Carbon Dioxide Extraction of Costus Oil	Inhibition zones: 12–15 mm	Ahmed et al. (2022)	
*Aspergillus niger*	ND	Powder	Ethanol ultra-sonicated for 40 min	Inhibition zones concentrations at 10–50 μL/mL 9.5–17 mm MIC: 0.3 mg/mL	Deabes et al. (2021)	
		Roots	Methanol and aqueous + Iron oxide nanoparticles	Inhibition zones: 5 mm	Al-brahim (2023)	
*Aspergillus flavus*	NRRL 3357	Powder	Ethanol ultra-sonicated for 40 min	Inhibition zones concentrations at 10–50 μL/mL 9.5–16.5 mm MIC: 0.3 mg/mL	Deabes et al. (2021)	​
	RCMB 002002	Roots	Supercritical Carbon Dioxide Extraction of Costus Oil	NA	Ahmed et al. (2022)	
*Aspergillus parasiticus*	SSWT 2999	Powder	Ethanol ultra-sonicated for 40 min	Inhibition zones concentrations at 10–50 μL/mL 11–16.5 mm MIC: 0.8 mg/mL	Deabes et al. (2021)	
*Aspergillus carbonarius*	ITAL 204	Powder	Ethanol ultra-sonicated for 40 min	Inhibition zones concentrations at 10–50 μL/mL 10.5–17 mm MIC: 0.9 mg/mL	Deabes et al. (2021)	
*Aspergillus ochraceus*	ITAL 14	Powder	Ethanol ultra-sonicated for 40 min	Inhibition zones concentrations at 10–50 μL/mL 12.5–22.5 mm MIC: 0.2 mg/mL	Deabes et al. (2021)	
*Fusarium verticillioides*	ITEM 10027	Powder	Ethanol ultra-sonicated for 40 min	Inhibition zones concentrations at 10–50 μL/mL 10.5–15.5 mm MIC: 0.6 mg/mL	Deabes et al. (2021)	
*Fusarium proliferatum*	MPVP	Powder	Ethanol ultra-sonicated for 40 min	Inhibition zones concentrations at 10–50 μL/mL 9.5–16.5 mm MIC: 1.2 mg/mL	Deabes et al. (2021)	
*Fusarium oxysporium*	RCMB008 001 “2”	Roots	Supercritical Carbon Dioxide Extraction of Costus Oil	NA	Ahmed et al. (2022)	
*Penicillium verrucosum*	ND	Powder	Ethanol ultra-sonicated for 40 min	Inhibition zones concentrations at 10–50 μL/mL 10.5–15.5 mm MIC: 0.6 mg/mL	Deabes et al. (2021)	
*Yersinia pestis*	ND	Roots	Methanol, ethanol, acetone	Inhibition zones: 14.66–23.66 mm	Sagar et al. (2017)	
*Alternaria sp*	ND	Roots	Methanol and aqueous + Iron oxide nanoparticles	NA	Al-brahim (2023)	
*Rhizopus* sp	ND	Roots	Methanol and aqueous + Iron oxide nanoparticles	NA	Al-brahim (2023)	
*Candida parapsilosis*	ND	Roots	Methanol n-Hexane, n-Butanol, Dichloromethane, and Ethyl acetate	Inhibition zones: 11–13 mm MIC: 1–3.5 mg/mL	Soliman et al. (2022)	​
*Candida pseudotropicalis*	ND	Roots	Methanol n-Hexane, n-Butanol, Dichloromethane, and Ethyl acetate	Inhibition zones: 0.8–15 mm MIC: 0.25–4.5 mg/mL	Soliman et al. (2022)	
*Candida guillimondii*	ND	Roots	Methanol n-Hexane, n-Butanol, Dichloromethane, and Ethyl acetate	Inhibition zones: 12–14 mm MIC: 1–3 mg/mL	Soliman et al. (2022)	
*Candida glabrata*	ND	Roots	Methanol MgO nanoparticles	Inhibition zones: 19 mm	Amina et al. (2020)	

ND (Not determined): The studies did not report the source of pathogenic microbe.

NA (No activity): The studies report that the extracts have no activity against the tested microbes.

### 3.3 Rhus tripartite

Six studies have examined the antimicrobial properties of *R. tripartita*, utilizing various plant parts including leaves, roots, and stems. The investigations included Gram-positive and Gram-negative bacteria, as well as fungal species (Mohammed, 2015; Barka et al., 2019; Habibi et al., 2015). The extraction techniques primarily employed solvent-based methods, with solvents such as methanol, ethanol, ethyl acetate, hexane, chloroform, and petroleum ether.

The Gram-positive bacterial strains evaluated included Methicillin-Resistant *Staphylococcus aureus* (MRSA), non-resistant *S. aureus*, *Bacillus licheniformis*, *Bacillus circulans*, *Bacillus megaterium*, *B. subtilis*, *K. varians*, *Listeria monocytogenes*, *Bacillus cereus*, and *Enterococcus faecalis* (Mohammed, 2015; Asmaa et al., 2020; Bereksi et al., 2018; Ben Barka et al., 2016; Habibi et al., 2015). *Rhus tripartita* extracts demonstrated antibacterial activity against the majority of these strains. Notably, the extracts exhibited MICs of 10.5 μg/mL and 11.2 μg/mL against MRSA and non-resistant *S. aureus*, respectively (Mohammed, 2015). Other studies confirmed susceptibility of *L. monocytogenes*, *B. cereus*, and *E. faecalis* to the extracts (Bereksi et al., 2018; Barka et al., 2019). However, susceptibility varied for other strains: while *B. circulans*, *B. megaterium*, and *B. subtilis* were sensitive to the extracts, *K. varians* and *B. licheniformis*did showed no observable activity (Asmaa et al., 2020).

The Gram-negative bacteria assessed included *P. aeruginosa*, *A. hydrophila*, *P. mirabilis*, *E. coli*, *K. pneumoniae*, *Entrobacter cloacae*, *Escherichia vulneris*, and *S. typhimurium* (Barka et al., 2019; Habibi et al., 2015; Bereksi et al., 2018; Ben Barka et al., 2016). *Pseudomonas aeruginosa*, *E. coli*, and *K. pneumoniae* were the most commonly tested, and all studies reported antibacterial effects of *R. tripartita* extracts against them, with MIC values ranging from 1.6 to 50 mg/mL. Extracts prepared using hydromethanol generally showed greater potency, reflected by lower MIC values—for instance, 12.8 mg/mL and 25–50 mg/mL against *P. aeruginosa*—compared to extracts obtained with chloroform, ethanol, or petroleum ether, which displayed MICs of 50 mg/mL (Bereksi et al., 2018; Habibi et al., 2015). In contrast, Asmaa et al., 2020) reported no activity of the extracts against *E. vulneris* (Asmaa et al., 2020).

Fungal strains were evaluated in only one study, which included *Candida albicans*, *A. niger*, and *A. flavus* (Barka et al., 2019). Methanolic extracts exhibited antifungal activity, with MIC values ranging from 12.5 to 25 mg/mL. The MBCs were also reported, ranging between 25 and 50 mg/mL (Table 7).

**TABLE 7:** Comprehensive analysis of *R. tripartita* reported *in vitro* studies: treatment protocols and observed effects.

Microbe name	Sources	Part used	Extraction method	Units	References	Commercial availability
Gram-positive						
*Staphylococcus aureus*	ATCC 29213	Leaves	Ethanol, Ethyl Acetate	11.2 mg/mL (non-MRSA)	Mohammed (2015)	Not available
		Roots and Aerial	Methanol; Hydromethanol	MIC: 0.2–0.8 mg/mL	Bereksi et al. (2018)	
		Roots	Methanol	Inhibition zones 14.33 mm MIC: 25 mg/mL MBC: 50 mg/mL	Ben Barka et al. (2016)	
*Methicillin Resistant Staphylococcus aureus*	ATCC 33591 (MRSA)	Leaves	Ethanol, Ethyl Acetate	MIC: 10.5 mg/mL (MRSA)	Mohammed (2015)	
*Bacillus licheniformis*	ND	Leaves	Methanol, Hexane, ethanol and chloroform	NA	Asmaa et al. (2020)	
*Bacillus circulans*	ND	Leaves	Methanol, Hexane, ethanol and chloroform	MIC: 240 μL/mL	Asmaa et al. (2020)	
*Bacillus megaterium*	ND	Leaves	Methanol, Hexane, ethanol and chloroform	MIC: 240 μL/mL	Asmaa et al. (2020)	
*Bacillus subtilis*	ND	Leaves	Methanol, Hexane, ethanol and chloroform	MIC: 650 μL/mL	Asmaa et al. (2020)	
*Kocuria varians*	ND	Leaves	Methanol, Hexane, ethanol and chloroform	NA	Asmaa et al. (2020)	
*Listeria monocytogenes*	ATCC 7644	Roots, Leaves, Stems	Methanol	MIC: 12.5–50 mg/mL MBC: 25–100 mg/mL	Barka et al. (2019)	
*Bacillus cereus*	ATCC1247	Roots, Leaves, Stems	Methanol	MIC: 25–50 mg/mL MBC: 50–100 mg/mL	Barka et al. (2019)	
*Enterococcus faecalis*	ATCC 29212	Aerial	Hydromethanol	6.4 mg/mL	Bereksi et al. (2018)	
Gram-Negative						
*Pseudomonas aeruginosa*	ATCC 27853	Roots, Leaves, Stems	Methanol; Hydromethanol	MIC: 25–50 mg/mL MBC: 50–100 mg/mL	Barka et al. (2019)	Not available
	ATTC27853	Leaves Aerial	Chloroform, ethanol, Petroleum Ether	MIC 50 mg/mL	Habibi et al. (2015)	
	ATCC 27853	Aerial	Hydromethanol	MIC: 12.8 mg/mL	Bereksi et al. (2018)	
*Escherichia coli*	ATCC 8739	Roots, Leaves, Aerial	Methanol	MIC: 25 mg/mL and 50 mg/mL	Ben Barka et al. (2016)	
	ATCC 25922	Aerial	Hydromethanol	MIC 3.4–12.8 mg/mL	Bereksi et al. (2018)	
	ATTC10412	Leaves	Chloroform, ethanol, Petroleum Ether	MIC: 50 mg/mL	Habibi et al. (2015)	
*Klebsiella pneumoniae*	ATCC13883	Roots, Leaves, Stems	Methanol; Hydromthanol	MIC: 6.5–12.5 mg/mL MBC: 12.5–25 mg/mL	Barka et al. (2019)	​
	ATCC 700603	Aerial	hydromethanolic	MIC: 1.6 mg/mL	Bereksi et al. (2018)	
*Entrobacter cloacae*	ATCC 13047	Aerial	Hydromethanol	MIC: 3.2–6.4 mg/mL	Bereksi et al. (2018)	
*Escherichia vulneris*	ND	Leaves	Methanol, Hexane, ethanol and chloroform	NA	Asmaa et al. (2020)	
*Salmonella typhimurium*	NCTC 6017	Roots	Methanol	Inhibition zones 26.33 mm MIC: 25 mg/mL MBC: 50 mg/mL	Ben Barka et al. (2016)	
*Salmonella aeruginosa*	ATCC 25922	Roots	Methanol	Inhibition zones 25 mm MIC: 25 mg/mL MBC: 50 mg/mL	Ben Barka et al. (2016)	
*Aeromonas hydrophila*	ND	Roots, Leaves, Stems	Methanol	MIC: 25–50 mg/mL MBC: 50–100 mg/mL	Barka et al. (2019)	
Fungi						
*Aspergillus flavus*	ND	Roots, Leaves, Stems	Methanol	MIC: 12.5 mg/mL, MBC:25 mg/mL	Barka et al. (2019)	Not available
*Aspergillus Niger*	ND	Roots, Leaves, Stems	Methanol	MIC: 12.5 mg/mL, MBC: 25 mg/mL	Barka et al. (2019)	
*Candida albicans*	ATCC 2091	Roots, Leaves, Stems	Methanol	MIC: 12.5–25 mg/mL MBC: 25–50 mg/mL	Barka et al. (2019)	

ND (Not determined): The studies did not report the source of pathogenic microbe.

NA (No activity): The studies report that the extracts have no activity against the tested microbes.

### 3.4 Chenopodium murale

Six studies have investigated the antimicrobial potential of *C. murale*, encompassing Gram-positive and Gram-negative bacteria, as well as fungal species (Abdel-Aziz et al., 2014; Parzhak Zoufan et al., 2026; Sellem et al., 2016). Various parts of the plant were utilized, including leaves, stems, flowers, roots, and the entire plant. Most studies employed solvent-based extraction methods, using a range of solvents such as methanol, ethanol, dichloromethane, chloroform, ethyl acetate, hexane, n-butanol, and cyclohexane. Only one study used a nanotechnology-based approach, synthesizing silver nitrite nanoparticles using aqueous leaf extracts of *C. murale* (Abdel-Aziz et al., 2014).

The Gram-positive bacterial strains evaluated included *S. aureus*, *B. subtilis*, *M. luteus*, *L. monocytogenes*, and *E. faecalis* (Parzhak Zoufan et al., 2026; Sellem et al., 2016; Aboukhalaf et al., 2020). *Staphylococcus aureus* was the most frequently tested organism; three studies reported inhibition zones ranging from 7 to 22 mm (Abdel-Aziz et al., 2014; Sellem et al., 2016; Parzhak Zoufan et al., 2026). However, two studies indicated either a lack of activity or enhanced susceptibility only when the extracts were incorporated into nanoparticles (Abdel-Aziz et al., 2014; Aboukhalaf et al., 2020). Extracts obtained via maceration using various solvents demonstrated notable antibacterial activity, with inhibition zones of 30.9 mm and 34.7 mm reported against *M. luteus* and *L. monocytogenes*, respectively (Sellem et al., 2016).

Gram-negative bacteria assessed in these studies included *P. aeruginosa*, *E. coli*, *E. aerogenes*, and *Salmonella typhimurium* (Parzhak Zoufan et al., 2026; Aboukhalaf et al., 2020; Sellem et al., 2016). Most strains were found to be susceptible to the extracts; however, *P. aeruginosa* showed resistance in one study (Aboukhalaf et al., 2020). In contrast, Sellem et al. (2016) reported significant antibacterial activity, with inhibition zones of 30.2 mm and 33.93 mm against *P. aeruginosa* and *E. aerogenes*, respectively (Sellem et al., 2016).

A majority of the studies also evaluated antifungal activity, testing a variety of fungal strains including *C. albicans*, *F. oxysporum* f. Sp. *Lycopersici*, *Cryptococcus neoformans*, and *C. tropicalis* (Sellem et al., 2016; Naqvi, 2023; Aboukhalaf et al., 2020)*. C. albicans* was the most frequently investigated fungal species; however, the reported results were variable. Sellem et al. (2016) observed antifungal activity against *C. albicans*, with an inhibition zone of 19.1 mm, whereas Aboukhalaf et al. (2020) reported no antifungal activity, including against *C. neoformans* (Sellem et al., 2016; Aboukhalaf et al., 2020). *Candida tropicalis* was found to be moderately susceptible, with one study reporting an inhibition zone of 5.1 mm (Table 8) (Sellem et al., 2016).

**TABLE 8:** Comprehensive analysis of *C. murale* reported *in vitro* studies: treatment protocols and observed effects.

Microbe	Sources	Part used	Extract method	Units	References	Commercial availability
Gram-positive						
*Staphylococcus aureus*	ND	Leaves	Aqueous leaf + silver nitrate nanoparticles	Aqueous extracts did not show any activity, and nanoparticles showed inhibition zones 5 mM	Abdel-Aziz et al. (2014)	Not available
	ATCC 6538	Leaves, Stem, Flowers	Cyclohexane, Dichloromethane, Ethyl Acetate, Aceton, Acetonitrile by maceration	Inhibition zones: 22.1 mm	Sellem et al. (2016)	
	ATCC6538	Roots and Flower	Methanol Ethanol	Inhibition zones Methanol extracts 7 mm at 2 mg/disc Ethanol extracts 13 mm at 2 mg/disc	Parzhak Zoufan et al. (2026)	
	ND	Whole plant	Ethanol Dichloromethane	NA	Aboukhalaf et al. (2020)	
*Bacillus subtilis*	ATCC6633	Roots and Flower	Methanol Ethanol	NA	Parzhak Zoufan et al. (2026)	
*Micrococcus luteus*	LB14110	Leaves, Stem, Flowers	Cyclohexane, Dichloromethane, Ethyl Acetate, Aceton, Acetonitrile by maceration	Inhibition zones: 30.9 mm	Sellem et al. (2016)	
*Listeria monocytogenes*	ATCC 19117	Leaves, Stem, Flowers	Cyclohexane, Dichloromethane, Ethyl Acetate, Aceton, Acetonitrile by maceration	Inhibition zones: 34.7 mm	Sellem et al. (2016)	
*Enterococcus faecalis*	ND	Whole plant	Ethanol Dichloromethane	Inhibition zones: 5 mm	Aboukhalaf et al. (2020)	
Gram-Negative						
*Pseudomonas aeruginosa*	ATCC 49189	Leaves, Stem, Flowers	Cyclohexane, Dichloromethane, Ethyl Acetate, Aceton, Acetonitrile by maceration	Inhibition zones: 30.2 mm	Sellem et al. (2016)	Not available
	ATCC9027	Roots and Flower	Methanol Ethanol	Inhibition zones Methanol extracts 7 mm at 4 mg/disc Ethanol extracts 11 mm at 2 mg/disc	Parzhak Zoufan et al. (2026)	
	ND	Whole plant	Ethanol Dichloromethane	NA	Aboukhalaf et al. (2020)	
*Escherichia coli*	ATCC25922	Aerial, Leaves, Stem, Flowers	Methanol Ethanol	Inhibition zones Methanol extracts 13 mm at 2 mg/disc Ethanol extracts 6 mm at 2 mg/disc	Parzhak Zoufan et al. (2026)	
	ND	Whole plant	Ethanol Dichloromethane	Inhibition zones: 5 mm	Aboukhalaf et al. (2020)	
*Enterobacter aerogenes*	ATCC 13048	Stems, Leaves and Flowers	Cyclohexane, Dichloromethane, Ethyl Acetate, Aceton, Acetonitrile by maceration	Inhibition zones: 33.93 mm	Sellem et al. (2016)	​
*Salmonella typhimurium*	ATCC 14028	Stems, Leaves and Flowers	Cyclohexane, Dichloromethane, Ethyl Acetate, Aceton, Acetonitrile	Inhibition zones: 25 mm	Sellem et al. (2016)	
Fungi						
*Candida albicans*	ATCC 10231	Stems, Leaves and Flowers	Cyclohexane, Dichloromethane, Ethyl Acetate, Aceton, Acetonitrile by maceration	Inhibition zones: 19.1 mm	Sellem et al. (2016)	Not available
	ND	Whole plant	Ethanol Dichloromethane	NA	Aboukhalaf et al. (2020)	
*Fusarium oxysporum f. Sp. Lycopers*	isolated from roots of an infected tomato plant	Stem	Methanol extracts and sub-fraction n-Hexane Aqueous Chloroform Ethyl acetate	IC_50_ values: n-Hexane: 1.562 mg/mL Chloroform: 1.562 mg/mL Ethyl Acetate: 12.5 mg/mL	Naqvi (2023)	
*Cryptococcus neoformans*	ND	Whole plant	Ethanol Dichloromethane	NA	Aboukhalaf et al. (2020)	
*Candida tropicalis*	R2 CIP 203	Stems, Leaves and Flowers	Cyclohexane, Dichloromethane, Ethyl Acetate, Aceton, Acetonitrile by maceration	Inhibition zones: 5.1 mm	Sellem et al. (2016)	

ND (Not determined): The studies did not report the source of pathogenic microbe.

NA (No activity): The studies report that the extracts have no activity against the tested microbes.

### 3.5 Pyrus communis

The antimicrobial efficacy of *P. communis* has been explored in five studies, targeting both Gram-positive and Gram-negative bacteria, as well as various fungal strains (Żbikowska et al., 2025; Kiran et al., 2020; Erbil et al., 2018; Mahmood et al., 2022). Studies have utilized different parts of the plant, including the leaves, peel, pulp, seeds, and most frequently, the fruit. Extraction methods in these studies predominantly involved polar solvent-based techniques (Kiran et al., 2020; Güven et al., 2006; Erbil et al., 2018). Additionally, one study employed a novel method by synthesizing silver nanoparticles from aqueous *P. communis* extracts combined with AgNO_3_ to assess enhanced antimicrobial performance (Mahmood et al., 2022).

A range of Gram-positive bacterial strains has been examined to evaluate the antimicrobial activity of *P. communis*, including *S. aureus*, *S. epidermidis*, *S. subtilis*, *E. faecalis*, *L. monocytogenes*, *B. subtilis*, *B. cereus*, *Bacillus megaterium*, *Bacillus licheniformis*, *M. luteus*, *Proteus vulgaris*, *S. faecalis*, *Yersinia enterocolitica*, and *Bacillus pumilus*. Among these, *S. aureus* emerged as the most commonly investigated species (Güven et al., 2006; Mahmood et al., 2022; Kiran et al., 2020; Erbil et al., 2018). The inhibition zones reported against *S. aureus* ranged from 9 to 40 mm across different studies. Notably, the extracts demonstrated no inhibitory effect on MRSA, as indicated by Żbikowska et al. (2025). The most pronounced antibacterial effect (40 mm) was observed in extracts augmented with silver nanoparticles synthesized using AgNO_3_, according to Mahmood et al. (2022). This enhanced activity was also evident in other species such as *S. epidermidis*, which exhibited an inhibition zone of 33 mm under the same treatment conditions. Similarly, the study by Güven et al. (2006) reported antibacterial effects against a variety of strains, including *L. monocytogenes*, *B. subtilis*, *B. cereus*, *M. luteus*, *P. vulgaris*, and *Y. enterocolitica*, whereas *S. faecalis*was found to be resistant to the tested extracts (Güven et al., 2006).

A wide spectrum of Gram-negative bacterial strains, these include *E. coli*, *P. aeruginosa*, *H. pylori*, *Pseudomonas syringae* pv. *Phaseolicola*, *P. syringae* pv. *Glycine*, *Haemophilus influenzae*, *P. lachrymans*, *Xanthomonas campestris* pv. *Vesicatoria*, *P. tobacco*, *P. syringae* pv. *Tomato*, *Salmonella* spp., *S. typhimurium*, *Xanthomonas campestris* pv. *Campestris*, *Enterobacter aerogenes*, *Aeromonas hydrophila*, *K. pneumoniae*, *P. fluorescens*, *P. gingeri*, *P. gladioli* pv. *Agricola*, *Brucella* spp., and *P. maltophila*. Particularly, *E. coli* and *P. aeruginosa* were the most frequently studied. For *E. coli*, reported inhibition zones ranged from 7.1 to 18.07 mm (Żbikowska et al., 2025; Erbil et al., 2018; Kiran et al., 2020; Güven et al., 2006). Comparable results were observed for *P. aeruginosa*, although one study indicated that even the nanoparticle-enhanced extracts failed to exhibit any inhibitory effect against this organism (Mahmood et al., 2022). In a broader screening study, Güven et al. (2006) assessed the antimicrobial potential of *P. communis* extracts across multiple Gram-negative strains, observing variable outcomes ranging from weak to moderate inhibition (Güven et al., 2006).

Out of the eleven total studies, only two extended their investigations to include fungal pathogens. The tested fungal species comprised *Candida albicans*, *Candida glabrata*, *Geotrichum graminis*, *Cladosporium herbarum*, *Rhodotorula rubra*, *Fusarium oxysporum*, *Fusarium moniliforme*, *Penicillium notatum*, *Aspergillus fumigatus*, *A. parasiticus*, *A. niger*, *A. citri*, *Yarrowia lipolytica*, and *Saccharomyces cerevisiae* (Mahmood et al., 2022; Erbil et al., 2018; Güven et al., 2006). Among these, *C. albicans* was the only species found to be susceptible to the extracts, although one study by Erbil et al. (2018) reported resistance (Erbil et al., 2018). All other fungal strains tested exhibited resistance to *P. communis* extracts (Table 9) (Güven et al., 2006).

**TABLE 9:** Comprehensive analysis of *P. communis* reported *in vitro* studies: treatment protocols and observed effects.

Microbes	Sources	Part used	Extraction method	Units	References	Commercial availability
Gram-positive						
*Staphylococcus aureus*	ATCC 25923, MRSA 112	Leaves	*Methanol 60%*	Inhibition zones: 9.8–12.2 mm MRSA was unsusceptible	Żbikowska et al. (2025)	Not available
	ND	Peel and seeds	Aqueous extracts + AgNO_3_	Inhibition zones: 40 mm	Mahmood et al. (2022)	
	ND	Peel and pulp	Ethyl Acetate, Hydroalcoholic, Aqueous	Inhibition Zone: 1–12 mm MIC: 12.5–50 mg/mL	Kiran et al. (2020)	
	ATTC 6538 T	Fruits	Ethyl Acetate	Inhibition zones: 9 mm	Güven et al. (2006)	
	ATCC 6538	Fruits	Aqueous	Inhibition zones: 15.580–16.470 mm	Erbil et al. (2018)	
*Staphylococcus epidermidis*	ND	Peel and seeds	Aqueous extracts + AgNO_3_	Inhibition zones: 33 mm	Mahmood et al. (2022)	
*Staphylococcus subtillis*	ND	Peel and pulp	Ethyl Acetate, Hydroalcoholic, Aqueous	Inhibition Zone: 2–10 mm MIC: 12.5–75 mg/mL	Kiran et al. (2020)	
*Enterococcus faecalis*	ATCC 25212, HLAR 78	Leaves	Methanol 60%	NA	Żbikowska et al. (2025)	
*Listeria monocytogenes*	ND	Fruits	Ethyl Acetate	Inhibition zones: 10 mm	Güven et al. (2006)	
*Bacillus subtilis*	21	Leaves	Methanol 60%	Inhibition zones: 7.6–8 mm	Żbikowska et al. (2025)	
	NRRL 744	Fruits	Ethyl Acetate	Inhibition zones: 8 mm	Güven et al. (2006)	
	ND	Fruits	Aqueous	Inhibition zones: 14.41–18.39 mm	Erbil et al. (2018)	
*Bacillus cereus*	ND	Peel and pulp	Ethyl Acetate, Hydroalcoholic, Aqueous	Inhibition Zone: 2–8 mm MIC: 12.5–50 mg/mL	Kiran et al. (2020)	
	NRRL B-3711	Fruits	Ethyl Acetate	Inhibition zones: 10 mm	Güven et al. (2006)	
*Bacillus megaterium*	DSM 32	Fruits	Aqueous	Inhibition zones: 14.19–18.44 mm	Erbil et al. (2018)	
*Bacillus licheniformis*	ND	Fruits	Aqueous	NA	Erbil et al. (2018)	
*Microccoccus luteus*	NRRL B-4375	Fruits	Ethyl Acetate	Inhibition zones: 10 mm	Güven et al. (2006)	
*Proteus vulgaris*	NRRL B-123	Fruits	Ethyl Acetate	Inhibition zones: 8 mm	Güven et al. (2006)	
*Streptococcocus feacalis*	NRRL B-14617	Fruits	Ethyl Acetate	NA	Güven et al. (2006)	
*Yersinia enterocolitica*	ND	Fruits	Ethyl Acetate	Inhibition zones: 12 mm	Güven et al. (2006)	
*Bacillus pumilus*	B122	Fruits	Ethyl Acetate	Inhibition zones: 9 mm	Güven et al. (2006)	
Gram-Negative						
*Escherichia coli*	ATCC 25922, ESBL 79	Leaves	*Methanol 60%*	Inhibition zones: 7.1–8.7 mm ESBL 79: NA	Żbikowska et al. (2025)	Not available
	ND	Peel and pulp	Ethyl Acetate, Hydroalcoholic, Aqueous	Inhibition Zone: 2–9 mm MIC: 25–75 mg/mL	Kiran et al. (2020)	
	ATCC 25922	Fruits	Ethyl Acetate	Inhibition zones: 8 mm	Güven et al. (2006)	
	ND	Fruits	Aqueous	Inhibition zones: 12.34–18.07 mm	Erbil et al. (2018)	
*P. aeruginosa*	ATCC 27853, 256	Leaves	*Methanol 60%*	Inhibition zones: 8.8–9.3 mm For 256 strain: 6.9–8 mm	Żbikowska et al. (2025)	
	ND	Peel and seeds	Aqueous extracts + AgNO_3_	NA	Mahmood et al. (2022)	
	ATCC 27853	Fruits	Ethyl Acetate	Inhibition zones: 8 mm	Güven et al. (2006)	
	ATCC 9027	Fruits	Aqueous	Inhibition zones: 14.48–20.14 mm	Erbil et al. (2018)	
*H. pylori*	ATCC 43504	Leaves	Methanol 60%	Inhibition zones: 8.7–10.5 mm	Żbikowska et al. (2025)	
*pseudomonas syringae pv. Phaseolicola*	NCPPB 52	Fruits	Ethyl Acetate	NA	Güven et al. (2006)	
*Pseudomonas syringae pv. Glycine*	PG1-T	Fruits	Ethyl Acetate	Inhibition zones: 8 mm	Güven et al. (2006)	
*Pseudomonas syringae*	−1	Fruits	Ethyl Acetate	Inhibition zones: 1 mm	Güven et al. (2006)	
*Haemophilus influenzae*	(NTHi) ATCC 49247, (NTHi 6)	Leaves	Methanol 60%	Inhibition zones: 7.9–8.9 mm	Żbikowska et al. (2025)	
*Pseudomonas lachrymans*	ND	Fruits	Ethyl Acetate	NA	Güven et al. (2006)	
*Xanthamonas campestris pv. Vesicatoria*	75–3	Fruits	Ethyl Acetate	Inhibition zones: 8 mm	Güven et al. (2006)	
*Pseudomonas tobacco*	8	Fruits	Ethyl Acetate	NA	Güven et al. (2006)	
*Pseudomonas syringae pv. Tomato*	32	Fruits	Ethyl Acetate	Inhibition zones: 14 mm	Güven et al. (2006)	
*Salmonella* spp	ND	Peel and seeds	Aqueous extracts + AgNO_3_	NA	Mahmood et al. (2022)	
*Salmonella typhimurium*	ND	Fruits	Ethyl Acetate	NA	Güven et al. (2006)	
*Xanthamonas campestris pv. Campestris*	NRRL-B1459	Fruits	Ethyl Acetate	Inhibition zones: 8 mm	Güven et al. (2006)	
*Enterobacter aerogenes*	NRRL B-3567	Fruits	Ethyl Acetate	Inhibition zones: 12 mm	Güven et al. (2006)	
	ND	Fruits	Aqueous	Inhibition zones: 13.43–18.42 mm	Erbil et al. (2018)	
*Aeromonas hydrophila*	ND	Fruits	Ethyl Acetate	Inhibition zones: 12 mm	Güven et al. (2006)	
*Klebsiella pneumoniae*	ND	Fruits	Ethyl Acetate	Inhibition zones: 7 mm	Güven et al. (2006)	​
	ND	Fruits	Aqueous	Inhibition zones: 13.31–17.24 mm	Erbil et al. (2018)	
*Pseudomonas fluorescens*	B 130	Fruits	Ethyl Acetate	NA	Güven et al. (2006)	
*Pseudomonas gingeri*	3,146	Fruits	Ethyl Acetate	NA	Güven et al. (2006)	
*Pseudomonas gladioli pv. Agricola*	RR3	Fruits	Ethyl Acetate	Inhibition zones: 8 mm	Güven et al. (2006)	
*Brucella* spp.	ND	Fruits	Ethyl Acetate	NA	Güven et al. (2006)	
*Pseudomonas maltophila*	ND	Fruits	Ethyl Acetate	Inhibition zones: 8 mm	Güven et al. (2006)	
Fungi						
*Candida Albicans*	ND	Peel and seeds	Aqueous extracts + AgNO_3_	Inhibition zones: 21–29 mm	Mahmood et al. (2022)	Not available
	ND	Fruits	Ethyl Acetate	Inhibition zones: 10 mm	Güven et al. (2006)	
	ND	Fruits	Aqueous	NA	Erbil et al. (2018)	
*Candida globrata*	ND	Fruits	Ethyl Acetate	Inhibition zones: 10 mm	Güven et al. (2006)	
*Geotrichum graminis*	ND	Fruits	Ethyl Acetate	NA	Güven et al. (2006)	
*Cladosporum herbarum*	ND	Fruits	Ethyl Acetate	NA	Güven et al. (2006)	
*Rhodotorula rubra*	NRRL Y-2505	Fruits	Ethyl Acetate	NA	Güven et al. (2006)	
*Fusarium oxysporum*	NRRL 5836	Fruits	Ethyl Acetate	NA	Güven et al. (2006)	
*Fusarium moniliforme*	NRRL 1866	Fruits	Ethyl Acetate	NA	Güven et al. (2006)	
*Penicillium notatum*	NRRL 807	Fruits	Ethyl Acetate	NA	Güven et al. (2006)	
*Aspergillus fumigatus*	NRRL 163	Fruits	Ethyl Acetate	NA	Güven et al. (2006)	
*Aspergillus parasiticus*	NRRL 2999	Fruits	Ethyl Acetate	NA	Güven et al. (2006)	
*Aspergillus niger*	NRRL 321	Fruits	Ethyl Acetate	NA	Güven et al. (2006)	
*Aspergillus citri*	ND	Fruits	Ethyl Acetate	NA	Güven et al. (2006)	
*Yarrowia lipolytica*	ND	Fruits	Aqueous	NA	Erbil et al. (2018)	
*Saccharomyces cerevisiae*	ND	Fruits	Aqueous	NA	Erbil et al. (2018)	

ND (Not determined): The studies did not report the source of pathogenic microbe.

NA (No activity): The studies report that the extracts have no activity against the tested microbes.

### 3.6 Argemone ochroleuca

Three studies have explored the antimicrobial potential of *A. ochroleuca*, assessing its activity against Gram-positive bacteria, Gram-negative bacteria, and fungi (Opletal et al., 2014; Hernández Soto et al., 2026; Reyes et al., 2011). All studies utilized the aerial parts of the plant, and extraction was performed using various solvents, including methanol, ethanol, hexane, and ethyl acetate.

The Gram-positive bacterial strains tested included *S. aureus*, *E. faecalis*, *S. hyicus*, *Staphylococcus epidermidis*, *B. subtilis*, and *Sarcina lutea*. The extracts exhibited antibacterial activity against all tested Gram-positive organisms, although the MIC values varied depending on the bacterial source. For instance, Opletal et al. (2014) reported MIC values of 128 μg/mL for *S. hyicus*, 256 μg/mL for *E. faecalis*, and 125 μg/mL for *S. aureus* (Opletal et al., 2014). Conversely, Reyes et al. (2011) observed higher MIC values in strains resistant to ampicillin, cefotaxime, and dicloxacillin: 250 μg/mL for *B. subtilis*, 500 μg/mL for *S. epidermidis*, and greater than 2000 μg/mL for *S. lutea* (Reyes et al., 2011).

The Gram-negative bacteria examined included *E. coli*, *P. aeruginosa*, *P. mirabilis*, *V. cholerae*, *E. agglomerans*, *E. aerogenes*, *S. typhi*, and *Yersinia enterocolitica* (Opletal et al., 2014; Reyes et al., 2011). The antibacterial efficacy of *A. ochroleuca* extracts against these organisms was variable. In the study by Opletal et al. (2014), MIC values were 1,024 μg/mL for *E. coli* and *P. mirabilis*, and 2048 μg/mL for *P. aeruginosa* (Opletal et al., 2014). Reyes et al. (2011), which included both clinical and reference strains, reported moderate activity against *V. cholerae* (MIC: 1,500 μg/mL), while no activity was observed against *E. coli*, *E. agglomerans*, *S. typhi*, and *Y. enterocolitica* (Reyes et al., 2011).

Fungal strains investigated included *C. albicans*, *Cryptococcus neoformans*, *Trichophyton mentagrophytes*, *A. niger*, *Fusarium moniliforme*, *Fusarium sporotrichum*, *Fusarium oxysporum*, *Rhizoctoina solani*, and *Colletotrichum gloeosporioides*. Reyes et al. (2011) found that only *C. albicans* and *C. neoformans* were susceptible to the methanolic extracts, with IC_50_ values of 1750 μg/mL and 500 μg/mL, respectively (Reyes et al., 2011). The same study reported no antifungal activity against *T. mentagrophytes*, *A. niger*, *F. moniliforme*, and *F. sporotrichum*. Another study that evaluated *F. oxysporum*, *R. solani*, and *C. gloeosporioides* recorded high MIC values of 5,877 mg/L, 5,845 mg/L, and 3,521 mg/L, respectively, indicating limited antifungal activity (Table 10) (Hernández Soto et al., 2026).

**TABLE 10:** Comprehensive analysis of reported *A. ochroleuca* *in vitro* studies: treatment protocols and observed effects.

Microbe	Sources	Part used	Extract method	Units	References	Commercial availability
Gram-positive						
*Staphylococcus aureus*	CCM3953	Aerial	Tertiary alkaloid extraction: ethanol, Ethyl Acetate and Methanol	MIC: 64 μg/mL	Opletal et al. (2014)	**Not available**
	ATCC 12398		Hexane, Ethyl Acetate and Methanol	Methanol extracts MIC 125 μg/mL MBC: 250 μg/mL	Reyes et al. (2011)	
*Enterococcus faecalis*	CCM4224	Aerial	Tertiary alkaloid extraction: ethanol, Ethyl Acetate and Methanol	MIC: 256 μg/mL	Opletal et al. (2014)	
*Staphylococcus hyicus*	CCM2368	Aerial	Tertiary alkaloid extraction: ethanol, Ethyl Acetate and Methanol	MIC: 128 μg/mL	Opletal et al. (2014)	
*Staphylococcus epidermidis*	Clinical Isolates resistant to ampicillin, cefotaxime, and dicloxacillin	Aerial	Hexane, Ethyl Acetate and Methanol	Methanol extracts MIC: 500 μg/mL	Reyes et al. (2011)	
*Bacillus subtilis*	Clinical Isolates resistant to ampicillin, cefotaxime, and dicloxacillin	Aerial	Hexane, Ethyl Acetate and Methanol	Methanol extracts MIC: 250 μg/mL	Reyes et al. (2011)	
*Sarcina lutea*	Clinical Isolates resistant to ampicillin, cefotaxime, and dicloxacillin	Aerial	Hexane, Ethyl Acetate and Methanol	Methanol extracts MIC: >2000 μg/mL	Reyes et al. (2011)	
Gram-Negative						
*Escherichia coli*	CCM3954	Aerial	Tertiary alkaloid extraction: ethanol, Ethyl Acetate and Methanol	MIC: 1,024 μg/mL	Opletal et al. (2014)	Not available
	ATCC 25922	Aerial	Hexane, Ethyl Acetate and Methanol	NA	Reyes et al. (2011)	
*Pseudomonas aeruginosa*	CCM3955	Aerial	Tertiary alkaloid extraction: ethanol, Ethyl Acetate and Methanol	MIC: 2048 μg/mL	Opletal et al. (2014)	
*Proteus mirabilis*	CCM7188	Aerial	Tertiary alkaloid extraction: ethanol, Ethyl Acetate and Methanol	MIC: 1,024 μg/mL	Opletal et al. (2014)	
*Vibrio cholerae*	INDRE 206 • Isolated from polluted water (clinical strain) • O1 group, Inaba serotype, “El Tor” biotype • Enterotoxin producer CDC V12 No-O1 Ampicillin resistant	Aerial	Hexane, Ethyl Acetate and Methanol	Methanol extracts MIC: 1,500 μg/mL	Reyes et al. (2011)	
*Enterobacter agglomerans*	ATCC 27155	Aerial	Hexane, Ethyl Acetate and Methanol	NA	Reyes et al. (2011)	
*Enterobacter aerogenes*	Clinical isolates resistant to cephalosporins and ampicillin	Aerial	Hexane, Ethyl Acetate and Methanol	NA	Reyes et al. (2011)	​
*Salmonella typhi*	ATCC 19430	Aerial	Hexane, Ethyl Acetate and Methanol	NA	Reyes et al. (2011)	
*Yersinia enterocolitica*	Clinical isolates Ampicillin resistant	Aerial	Hexane, Ethyl Acetate and Methanol	NA	Reyes et al. (2011)	
Fungi						
*Candida albican*	ATCC 14065, ATCC 10231, and a clinical strain	Aerial	Hexane, Ethyl Acetate and Methanol	Methanol extracts IC_50_: 1750 μg/mL MBC: 3,500 μg/mL	Reyes et al. (2011)	Not available
*Cryptococcus neoformans*	ND	Aerial	Hexane, Ethyl Acetate and Methanol	Methanol extracts IC_50_: 500 μg/mL	Reyes et al. (2011)	
*Trichophyton mentagrophytes*	ND	Aerial	Hexane, Ethyl Acetate and Methanol	NA	Reyes et al. (2011)	
*Aspergillus niger*	ND	Aerial	Hexane, Ethyl Acetate and Methanol	NA	Reyes et al. (2011)	
*Fusarium moniliforme*	ND	Aerial	Hexane, Ethyl Acetate and Methanol	NA	Reyes et al. (2011)	
*Fusarium sporotrichum*	ATCC NRRL 3299	Aerial	Hexane, Ethyl Acetate and Methanol	NA	Reyes et al. (2011)	
*Fusarium oxysporum*	Isolated from plants	Aerial	Methanol by maceration	MIC: 5,877 mg/L	Hernández Soto et al. (2026)	
*Rhizoctoina solani*	Isolated from plants	Aerial	Methanol by maceration	MIC: 5,845 mg/L	Hernández Soto et al. (2026)	
*Colletotrichum gloeosporioide*	Isolated from plants	Aerial	Methanol by maceration	MIC: 3,521 mg/L	Hernández Soto et al. (2026)	

ND (Not determined): The studies did not report the source of pathogenic microbe.

NA (No activity): The studies report that the extracts have no activity against the tested microbes.

### 3.7 Trigonella hamosa

Two studies have evaluated the antimicrobial properties of *T*. *hamosa*, specifically its antibacterial activity against both Gram-positive and Gram-negative bacteria, as well as its antifungal potential (Sameer and Nayer, 2017; Rao et al., 2023). Both studies utilized the aerial parts of the plant. The extraction procedures predominantly employed solvent-based methods, using methanol, n-hexane, chloroform, n-butanol, and dichloromethane as solvents.

The Gram-positive bacterial strains tested included *S*. *aureus*, *B*. *subtilis*, *E*. *faecalis*, *Bacillus pumilus*, *S*. *epidermidis*, and *M*. *luteus*. In the study by Sameer and Nayer (2017), the extracts demonstrated antibacterial activity against *B. subtilis* and *E. faecalis*, with inhibition zones of 14 mm and 8 mm, and MICs of 7 mg/mL and 8.5 mg/mL, respectively, and no activity was observed against *S. aureus* (Sameer and Nayer, 2017). In contrast, the study by Rao et al. (2023) reported broader activity against *S. aureus*, *B. subtilis*, *E. faecalis*, *B. pumilus*, *S. epidermidis*, and *M. luteus*, with inhibition zones ranging from 14 to 20 mm at a concentration of 1 mg/mL (Rao et al., 2023).

Regarding Gram-negative bacteria, only three strains were assessed: *E. coli*, *P. aeruginosa*, and *Bordetella bronchiseptica*. Similar to the Gram-positive findings, Sameer and Nayer (2017) observed no inhibitory effect of the extracts on these strains (Sameer and Nayer, 2017). However, Rao et al. (2023) reported measurable activity, with inhibition zones between 12 mm and 17 mm at the same extract concentration (1 mg/mL) (Rao et al., 2023).

The antifungal activity of *T. hamosa* was tested against *C. albicans*, *A*. *niger*, and *Fusarium* species. Among these, only *C. albicans* was susceptible to the extracts, with an inhibition zone of 16 mm and an MIC of 5.5 mg/mL. *Aspergillus niger* and *Fusarium* spp. Did not exhibit sensitivity to the extracts (Table 11) (Sameer and Nayer, 2017).

**TABLE 11:** Comprehensive analysis of *Trigonella hamosa* reported *in vitro* studies: treatment protocols and observed effects.

Microbe	Sources	Part used	Extract method	Units	References	Commercial availability
Gram-positive						
*Staphylococcus aureus*	ATTC 25923	Aerial	Methanol solution, (Methanol:Dichloromethane 1:1 v/v)	NA	Sameer and Nayer (2017)	Not available
	ND		Methanol, n-Hexane, chloroform, and n-Butanol	Inhibition zone at concentration 1 mg/mL:14 mm	Rao et al. (2023)	
*Bacillus subtilis*	ATCC 6633		Methanol solution, (Methanol:Dichloromethane 1:1 v/v)	Inhibition zones: 14 mm MIC: 7 mg/mL	Sameer and Nayer (2017)	
	ND		Methanol, n-Hexane, chloroform, and n-Butanol	Inhibition zones at concentration 1 mg/mL: 20 mm	Rao et al. (2023)	
*Enterococcus faecalis*	ATCC 29212		Methanol solution, (Methanol:Dichloromethane 1:1 v/v)	Inhibition zones: 13 mm MIC: 8.5 mg/mL	Sameer and Nayer (2017)	
*Bacillus pumilus*	ND		Methanol, n-Hexane, chloroform, and n-Butanol	Inhibition zone at concentration 1 mg/mL: 16 mm	Rao et al. (2023)	
*Staphylococcus epidermidis*				Inhibition zone at concentration 1 mg/mL: 18 mm		
*Micrococcus luteus*				Inhibition zone at concentration 1 mg/mL: 16 mm		
Gram-Negative						
*Escherichia coli*	ATCC 8739	Aerial	Methanol solution, (Methanol:Dichloromethane 1:1 v/v)	NA	Sameer and Nayer (2017)	Not available
	ND		Methanol, n-Hexane, chloroform, and n-Butanol	Inhibition zone at concentration 1 mg/mL: 17 mm	Rao et al. (2023)	
*Pseudomonas aeruginosa*	ATCC 4027		Methanol solution, (Methanol:Dichloromethane 1:1 v/v)	NA	Sameer and Nayer (2017)	
	ND		Methanol, n-Hexane, chloroform, and n-Butanol	Inhibition zone at concentration 1 mg/mL: 12 mm	Rao et al. (2023)	
*Bordetella bronchiseptica*				NA		
Fungi						​
*Candida albicans*	ATCC10231	Aerial	Methanol solution, (Methanol:Dichloromethane 1:1 v/v)	Inhibition zones: 16 mm MIC: 5.5 mg/mL	Sameer and Nayer (2017)	Not available
*Fusarium sp*	ND			NA		
*Aspergillus niger*						

ND (Not determined): The studies did not report the source of pathogenic microbe.

NA (No activity): The studies report that the extracts have no activity against the tested microbes.

### 3.8 Galium odoratum

**FIGURE 3:**
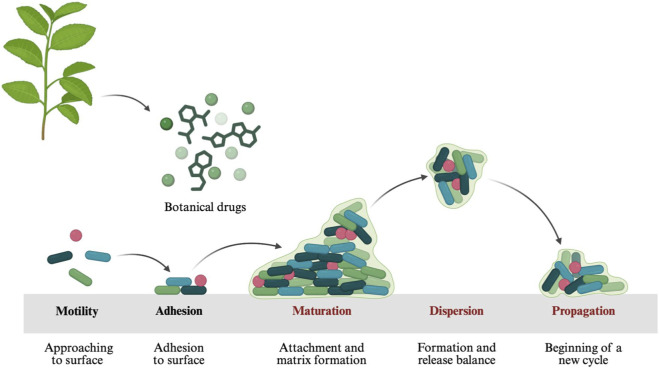
Schematic representation of the potential antibiofilm activity of botanical drugs during different stages of biofilm development, including motility, adhesion, maturation, dispersion, and propagation. Botanical compounds may inhibit biofilm formation by interfering with microbial attachment, matrix formation, and cell dispersal.

Only a single study has been identified that examined the antibacterial activity of *G. odoratum*, focusing specifically on its leaf metabolites (Wojnicz et al., 2012). The plant material was extracted using a solvent-based method, and the investigation targeted *E. coli*. Notably, the study suggested a potential mechanism of action, proposing that the plant extract exerts an anti-biofilm effect by altering bacterial surface structures involved in adhesion to surfaces (Table 12). The proposed mechanism is illustrated in Figure 3.

**TABLE 12:** Comprehensive analysis of reported *G. odoratum in vitro* studies: treatment protocols and observed effects.

Microbe name	Sources	Part used	Extraction method	Units	Proposed mechanisms	References	Commercial availability
*Escherichia coli*	Clinical strain isolated from the urine of patient with pyelonephritis	Leaves	Hot water extraction	20 mg/mL reduce the viability to less than 60%	Anti-biofilm effect of plant extracts can be caused by modifications in the bacterial surface structures responsible for binding to the occupied surface	Wojnicz et al. (2012)	Not available

## 4 Discussion

This systematic review highlights the diverse antimicrobial potential of the selected medicinal plants, reflecting a growing global interest in plant-derived bioactive metabolites as alternatives or adjuncts to conventional antimicrobial agents in the face of escalating AMR. Across the included *in vitro* and *in vivo* studies, *L. sativum, S. costus, P. communis, C. murale, R. tripartita, T. hamosa, A. ochroleuca, and G. odoratum* demonstrated varying degrees of antibacterial and antifungal activity against a broad spectrum of Gram-positive, Gram-negative, and fungal pathogens. Notably, *L. sativum* and *S. costus* were repeatedly shown to inhibit clinically relevant pathogens such as *S. aureus*, *E. coli*, and *C*. *albicans*, with enhanced efficacy often observed when extracts were incorporated into nanoparticle formulations. These findings support the longstanding traditional medicinal use of these species in treating infectious diseases and provide a rationale for advancing their evaluation in standardized preclinical and clinical models.

The extensive investigation of *L. sativum* in the context of its antibacterial and antifungal properties underscores the plant’s potential as a source of natural antimicrobial agents. The MIC values reported by Ait-Yahia et al. (2018) suggest an efficacy that may depend on both the solvent used for extraction and the specific bacterial strain being tested. The notable antibacterial effects of *L. sativum* extracts, especially against *S. aureus*, suggest that the plant could play a role in the development of new treatments for infections caused by antibiotic-resistant strains. However, the resistance exhibited by *P*. *mirabilis*, even against nanoparticle formulations, highlights the challenges that remain in harnessing *L. sativum’s* antimicrobial properties fully. Alternative extraction methods, such as the synthesis of silver nanoparticles, represents a promising approach for enhancing the antimicrobial efficacy of *L. sativum.* The increased inhibition zones observed with nanoparticle formulations, as reported by Haj Bloukh et al. (2021) and Alnehia et al. (2023), suggest that these approaches could amplify the bioactivity of plant extracts, potentially leading to more effective treatments. In addition to its antibacterial properties, the antifungal activity of *L. sativum* has been a focal point of research, particularly against *C. albicans*, a common pathogen associated with opportunistic infections. The positive antifungal effects reported across multiple studies indicate that *L. sativum* could serve as a valuable resource for developing antifungal therapies. However, the lack of antifungal activity observed in the study by Rezig et al. (2022) underscores the necessity for further investigation into the conditions that influence the antifungal efficacy of *L. sativum* extracts. The available studies on the antimicrobial activity of Lepidium sativum indicate promising antibacterial potential, particularly from seed extracts; however, significant methodological limitations remain. Approximately 50% (10/20) relied solely on disc diffusion assays without incorporating MIC or MBC determinations, limiting the quantitative assessment of antimicrobial potency. In contrast, only about 47% of studies included MIC analysis, while fewer studies additionally evaluated MBC values. Seeds were the most frequently investigated plant part, accounting for nearly 80% (16/20) of studies, whereas leaves were examined in only a small proportion of investigations. Furthermore, mechanistic evaluation was notably limited, with approximately 75% (15/20) of studies failing to propose or experimentally investigate mechanisms of antimicrobial action. Although phytochemical characterization has improved in recent years, around 30% (6/20) of studies did not include any phytochemical analysis, while several others performed only qualitative screening. Considerable variability was also observed in extract concentrations, which ranged from microgram to gram-level doses, reflecting poor methodological standardization across studies. Collectively, these findings suggest that despite growing evidence supporting the antimicrobial potential of *L. sativum*, the current literature remains constrained by methodological inconsistencies, insufficient mechanistic validation, and limited phytochemical–bioactivity correlation.

For *S. costus*, the studies reviewed indicate an antimicrobial activity of *S. costus* extracts against Gram-positive, Gram-negative bacteria, and fungi. The observed inhibition zones suggest that *S. costus* possesses bioactive metabolites capable of disrupting bacterial growth. This finding is particularly relevant given the increasing prevalence of antibiotic-resistant strains of *S. aureus*, including MRSA. The choice of solvent appears to influence the extraction efficiency of the bioactive metabolites, as polar solvents such as methanol and ethanol yielded the most promising results. The use of innovative extraction methods, such as supercritical carbon dioxide extraction, resulted in markedly higher antibacterial efficacy. This underscores the importance of optimizing extraction techniques to enhance the yield and potency of antimicrobial agents derived from plant sources. While some formulations demonstrated comparable antibacterial activity to crude extracts, others, such as methanol-based magnesium oxide nanoparticles, exhibited selective efficacy. Regarding antifungal properties, the activity observed in studies utilizing various solvent extractions is promising; however, the variability with nanoparticle formulation results highlights the need for more research.

Among the reviewed studies on the antimicrobial activity of *S. costus*, approximately 54% (7/13) relied solely on disc diffusion assays without MIC or MBC/MFC determination, limiting quantitative evaluation of antimicrobial potency. Roots were the dominant plant part investigated in nearly 92% (12/13) of studies, while mechanistic investigations were limited, with about 77% (10/13) of studies failing to experimentally assess mechanisms of action. Although phytochemical characterization was comparatively well addressed, with approximately 77% (10/13) of studies employing GC-MS, HPLC, FTIR, or related analytical techniques, few studies correlated identified compounds with antimicrobial activity. Considerable variation in extraction methods and concentration reporting further highlights the lack of methodological standardization across the current literature.

*Pyrus communis* showed variabilities in the antibacterial results; this variability may be attributed to differences in extraction methods, concentrations of plant extracts, and the specific strains of *S. aureus* tested. Interestingly, while *P. communis* extracts demonstrated significant antimicrobial activity against *S. aureus*; however, they were ineffective against MRSA, as reported by Żbikowska et al. (2025). The enhanced antimicrobial effects observed when combining *P. communis* extracts with silver nanoparticles, as demonstrated by Mahmood et al. (2022), represent a promising antimicrobial property for future research. The pronounced inhibition zones against *S. aureus* and *S. epidermidis* suggest that such synergistic approaches may enhance the efficacy of plant-derived antimicrobials. However, it is noteworthy that even with nanoparticle enhancement, extracts failed to exhibit inhibitory effects against *P. aeruginosa* in one study (Mahmood et al., 2022). In addition, the resistance exhibited by other fungal strains, including *Aspergillus* species, suggests that the antifungal potential of *P. communis* may be limited. The studies relied solely on disc diffusion assays without MIC or MBC determination, limiting quantitative assessment of antimicrobial potency. Various plant parts, including peels, pulp, seeds, fruits, and leaves, were investigated, indicating broader plant utilization compared with several other medicinal plants. However, mechanistic investigations were absent in all reviewed studies. Phytochemical characterization was moderately represented, with approximately 60% (3/5) of studies including FTIR, TPC/TFC, or spectrophotometric analyses, although direct correlations between phytochemicals and antimicrobial activity were generally lacking.

*Chenopodium murale* has a pronounced antibacterial activity observed against Gram-positive bacteria, particularly *S. aureus*, which suggests the potential of *C. murale* as a therapeutic agent. However, inconsistent results in efficacy against Gram-negative bacteria, especially the resistance noted in *P. aeruginosa*, indicate that further exploration is required to elucidate the mechanisms of action and resistance. The only study employing a nanotechnology approach demonstrated a novel avenue for enhancing antimicrobial effectiveness, suggesting that future research could benefit from integrating such methodologies. The antifungal activity revealed conflicting results across studies, which highlights the need for further exploration of factors influencing antimicrobial activity. The available studies depended on disc diffusion assays, while the less studies incorporated MIC and MBC evaluations for quantitative antimicrobial assessment. Investigations into mechanisms of action were scarce, with just one study examining antibiofilm effects and motility inhibition, whereas the majority did not explore antimicrobial mechanisms. Phytochemical profiling was more frequently addressed, as most studies conducted analyses such as GC-MS, TPC, TFC, or qualitative phytochemical screening. Nonetheless, significant inconsistencies in extraction concentrations, plant materials used, and experimental methodologies continue to limit the reproducibility and comparability of the reported findings.

*Rhus tripartita* studies comprehensively examined the efficacy of various plant parts, leaves, roots, and stems, which offer a comprehensive and integrative reflection of the bioactivity. The studies reviewed have predominantly utilized solvent-based extraction methods, and notably, hydromethanol extracts demonstrated enhanced potency, yielding lower MICs against several bacterial strains. Also, *R. tripartita* extracts exhibited notable activity against MRSA and non-resistant *S. aureus*. Furthermore, the susceptibility of other Gram-positive strains, such as *L. monocytogenes*, *B. cereus*, and *E. faecalis*, further underscores the potential utility of *R. tripartita* in developing alternative antimicrobial therapies. However, the variability in susceptibility among different bacterial strains, as observed with *B. licheniformis* and *K. varians*, emphasizes the need for further investigation. *Rhus tripartita* demonstrated promising antimicrobial potential, particularly against *P. aeruginosa*, *E. coli*, and *K. pneumoniae*. Hydromethanolic extracts consistently exhibited superior efficacy compared to those obtained using chloroform, ethanol, or petroleum ether, underscoring the influence of extraction methods on bioactivity. Antifungal investigations, though limited, revealed that methanolic extracts inhibited the growth of *C. albicans*, *A. niger*, and *A. flavus*. These findings highlight *R. tripartita* as a potential source of both antibacterial and antifungal agents, warranting further research across a broader spectrum of pathogens.

Studies on *R. tripartita* primarily used disc diffusion assays, with only two studies using MIC and MBC. All the included studies failing to experimentally assess modes of antimicrobial action. Phytochemical characterization was inconsistently performed; two studies did not include phytochemical analysis, while others mainly focused on total phenolic and flavonoid contents. Considerable gaps were also observed in reporting plant parts and extract concentrations, limiting reproducibility and comparative analysis across studies.

The antimicrobial properties of *T. hamosa*, as evaluated in two distinct studies, reveal a complex profile of activity against various bacterial and fungal strains. Notably, while Sameer and Nayer (2017) reported limited antibacterial effects, particularly against Gram-positive bacteria, Rao et al. (2023) demonstrated a broader spectrum of activity, suggesting variability in extraction techniques or phytochemical composition. The absence of activity against certain Gram-negative strains in the earlier study raises questions about the efficacy of the extracts and emphasizes the need for standardized methodologies. Furthermore, the antifungal potential against *C. albicans* highlights *T. hamosa*’s therapeutic promise, warranting further exploration into its bioactive metabolites and mechanisms of action. The antimicrobial studies on *T. hamosa* were limited in number and relied primarily on disc diffusion assays, with only one of the two reviewed studies including MIC determination. Neither study investigated mechanisms of antimicrobial action. However, both studies included some level of phytochemical characterization through GC-MS or qualitative phytochemical screening. Despite these findings, the current evidence remains insufficient due to the small number of studies and the absence of mechanistic and standardized antimicrobial evaluations.

The antimicrobial potential of *A. ochroleuca* has been systematically evaluated across three studies. The consistent antibacterial activity observed against Gram-positive bacteria, particularly strains such as *S. aureus* and *E. faecalis*, suggests that *A. ochroleuca* may harbor bioactive metabolites with significant therapeutic potential. However, the variability in MIC values highlights the influence of both the bacterial strain and the extraction method on antimicrobial efficacy. For example, the higher MIC values recorded for resistant strains by Reyes et al. (2011) underscore the challenge of combating antibiotic-resistant pathogens using plant-derived extracts. In contrast, the results for Gram-negative bacteria indicate a more limited efficacy, with notably high MIC values for strains like *P. aeruginosa* and *E. coli*. Fungal susceptibility was also limited, with only *C. albicans* and *C. neoformans* demonstrating appreciable sensitivity to the extracts. The high IC_50_ values reported for other fungal strains indicate that the antifungal potential of *A. ochroleuca* may be restricted, necessitating further research to explore the conditions under which its antifungal properties might be enhanced. The available studies on *A. ochroleuca* remain limited, with two studies employing disc diffusion assays and only one study incorporating MIC and MBC analyses. None of the reviewed studies investigated mechanisms of antimicrobial action. Phytochemical characterization was inconsistently addressed, with one study lacking phytochemical analysis entirely, while others employed GC-MS or thin-layer chromatography. Additionally, variability in concentration reporting and limited investigation of plant parts restrict the comparability and translational relevance of the available findings.

The limited research on the antibacterial properties of *G. odoratum*, particularly the study by Wojnicz et al. (2012), underscores the need for further investigation into its bioactive metabolites. The proposed mechanism of action, involving alterations to bacterial surface structures, opens opportunities for exploring the therapeutic potential of *G. odoratum* in combating biofilm-associated infections, warranting more comprehensive studies.

An important observation across the included studies for all nine plants is that the investigated formulations, primarily solvent-based extracts and nanoparticle-based systems, remain at the experimental stage, with no reported commercial availability. This highlights a significant gap between laboratory findings and real-world application, suggesting that despite promising antimicrobial activity, further research is required to address challenges related to standardization, stability, safety, and regulatory approval before these formulations can be translated into clinically or industrially viable products.

While this review demonstrates the promising antimicrobial activity of the selected medicinal plants, it is important to contextualize these findings within the broader landscape of antimicrobial therapy, particularly in the face of the growing global burden of AMR (Organization, 2025). Compared to synthetic antimicrobial agents, plant-derived formulations remain underrepresented in clinical practice despite increasing evidence of their *in vitro* efficacy. Conventional antibiotics typically consist of single, well-characterized active metabolites with standardized dosing and well-established pharmacokinetic profiles (Newman and Cragg, 2020). In contrast, plant-based extracts are complex mixtures of bioactive metabolites that may exert synergistic effects on multiple microbial targets, potentially enhancing efficacy and reducing the likelihood of resistance development (Wagner and Ulrich-Merzenich, 2009).

However, this complexity also presents challenges. Variability in phytochemical composition due to differences in plant source, extraction methods, and preparation techniques can lead to inconsistent antimicrobial activity, as reflected in the studies included in this review (Ekor, 2014). Furthermore, while several plant extracts demonstrated activity against clinically relevant pathogens such as *Staphylococcus aureus* and *E. coli*, their efficacy remains largely confined to *in vitro* settings, with limited validation in vivo or clinical models (Cowan, 1999). Therefore, although natural antimicrobial formulations derived from these plants show potential as alternative or adjunct therapeutic agents, significant gaps remain in standardization, safety evaluation, and clinical translation when compared to synthetic antimicrobials.

This review is distinguished from previous studies for several reasons. First, it is specifically tailored to medicinal plants relevant to the Middle Eastern context. Second, it provides a comprehensive evaluation of nine selected plants. Third, the review synthesizes key findings for each plant, offering a concise and accessible overview of their current stage of research on these selected plants and utilization, thereby facilitating future investigations in this field.

Several limitations should be acknowledged when interpreting the findings of this review. First, the majority of included studies relied on *in vitro* assays, which, while valuable for initial screening, do not account for pharmacokinetics, bioavailability, or potential toxicity *in vivo*. Second, considerable variability existed in extraction methods, plant parts used, and solvent polarity, making direct comparisons of antimicrobial potency challenging. Third, many studies lacked standardization in reporting outcomes, with inconsistent use of MIC values versus inhibition zone diameters, and limited inclusion of reference antibiotics for benchmarking activity. Additionally, the geographical sourcing of plant materials and environmental growth conditions, which can influence phytochemical composition, were often underreported. Finally, the literature search was conducted using Google Scholar, which may have led to the omission of relevant studies indexed exclusively in other scientific databases.

## 5 Conclusion

In conclusion, the emergence of antimicrobial resistance poses a significant threat to global health, necessitating the exploration of novel therapeutic agents. This systematic review showed that the selected medicinal plants exhibit promising antimicrobial properties against a variety of clinically significant bacterial and fungal pathogens, with some species demonstrating enhanced effects when formulated using nanotechnology-based approaches. These findings underscore the potential of ethnobotanically important plants as sources for novel antimicrobial agents in the fight against AMR. However, future research should prioritize standardized extraction and testing protocols, *in vivo* validation, and mechanistic studies to better characterize active metabolites and optimize their therapeutic potential. Such efforts will be essential for translating these preliminary observations into safe, effective, and sustainable antimicrobial therapies.

## Data Availability

The original contributions presented in the study are included in the article/Supplementary Material, further inquiries can be directed to the corresponding author.
