# Supplementary material for: Antimicrobial potential of selected medicinal plants against drug-resistant pathogens: a systematic review
Source: Front Pharmacol. 2026 Jul 9;17:1735625. doi: 10.3389/fphar.2026.1735625 (PMC13391537; doi:10.3389/fphar.2026.1735625)
Supplement: Supplementary file 1 [file Table1.docx]

**Table 1S. Homotypic and heterotypic synonyms of selected plant species used in the study**.

| **Plant** | **Synonyms** |
| --- | --- |
| ***Lepedium sativum*** | **Homotypic Synonyms**   - *Cardamon sativum* (L.) Fourr. - *Lepia sativa* (L.) Desv. - *Nasturtium sativum* (L.) Moench - *Thlaspi sativum* (L.) Crantz - *Thlaspidium sativum* (L.) Spach   **Heterotypic Synonyms**   - *Arabis chinensis* Rottler ex Wight - *Crucifera nasturtium* E.H.L. Krause - *Lepidium sativum* var. *australe* Alef. - *Lepidium sativum* var. *crispum* (Medik.) DC. - *Lepidium sativum* var. *latifolium* DC. - *Lepidium sativum* var. *pallidum* Alef. - *Lepidium sativum* var. *schimperi* Thell. - *Lepidium sativum* var. *silvestre* Thell. - *Lepidium sativum* subsp. *spinescens* (DC.) Thell. - *Lepidium sativum* var. *spinescens* (DC.) Jafri - *Lepidium sativum* var. *triplocrispum* Alef. - *Lepidium sativum* var. *vulgare* Alef. - *Lepidium spinescens* DC. - *Nasturtium crispum* Medik. - *Nasturtium hortense* Garsault - *Nasturtium spinescens* (DC.) Kuntze - *Nasturtium spinosum* Desv. ex Thell. - *Thlaspi nasturtium* Bergeret ex Steud. |
| ***Saussurea costus*** | **Homotypic Synonyms**   - *Aucklandia costus* Falc. - *Saussurea costus* (Falc.) Lipsch. - *Theodorea costus* (Falc.) Kuntze   **Heterotypic Synonyms**   - *Aplotaxis lappa* Decne. - *Aucklandia lappa* (Decne.) Decne. - *Saussurea lappa* (Decne.) Sch.Bip. |
| ***Rhus tripartita*** | **Homotypic Synonyms**   - *Rhus tripartita* (Ucria) Grande - *Rhamnus tripartita* Ucria - *Ziziphus tripartita* (Ucria) Schult.   **Heterotypic Synonyms**   - *Rhus albicans* Willd. - *Rhus crataegiformis* Pers. - *Rhus dioica* Brouss. ex Willd. - *Rhus oxyacantha* Schousb. ex Cav. - *Rhus oxyacantha* var. *ballii* Maire - *Rhus oxyacantha* var. *ziziphina* (Tineo) Ball - *Rhus oxyacanthoides* Dum.Cours. - *Rhus syriaca* Boiss. & Balansa - *Rhus ziziphina* Tineo - *Toxicodendron oxyacanthum* (Schousb. ex Cav.) Kuntze |
| ***Chenopodium murale*** | **Homotypic Synonyms**   - *Anserina muralis* (L.) Montandon - *Atriplex muralis* (L.) Crantz - *Chenopodium murale* L.   **Heterotypic Synonyms**   - *Chenopodium biforme* Nees - *Chenopodium carthagenense* Zuccagni - *Chenopodium carthagenense* Zuccagni - *Chenopodium chamrium* Buch.-Ham. - *Chenopodium congestum* Hook.f. - *Chenopodium flavum* Forssk. - *Chenopodium gandhium* Buch.-Ham. - *Chenopodium guineense* Jacq. - *Chenopodium ilicifolium* Griff. - *Chenopodium laterale* Aiton - *Chenopodium longidjawense* Peter - *Chenopodium lucidum* Gilib. - *Chenopodium maroccanum* Pau - *Chenopodium murale* var. *acutidentatum* Aellen - *Chenopodium murale* var. *albescens* Moq. - *Chenopodium murale* f. *albescens* (Moq.) Maire - *Chenopodium murale* var. *angustatum* Fenzl - *Chenopodium murale* var. *biforme* (Nees) Moq. - *Chenopodium murale* var. *carthagenense* Moq. - *Chenopodium murale* var. *latifolium* Fenzl - *Chenopodium murale* var. *microphyllum* Coss. & Germ. - *Chenopodium murale* var. *paucidentatum* Beck - *Chenopodium murale* var. *prostratum* A. Zobel - *Chenopodium murale* var. *spissidentatum* Murr - *Rhagodia baccata* var. *congesta* (Hook.f.) Hook.f. - *Rhagodia billardierei* var. *congesta* (Hook.f.) Benth. - *Rhagodia congesta* (Hook.f.) Moq. - *Vulvaria trachisperma* Bubani |
| ***Pyrus communis*** | Homotypic Synonyms   - *Malus communis* (L.) Poir. - *Sorbus pyrus* Crantz |
| *Argemone ochroleuca* | **Homotypic Synonyms**   - *Argemone mexicana* subsp. *ochroleuca* (Sweet) Schwarzb. - *Argemone mexicana* var. *ochroleuca* (Sweet) Lindl.   **Heterotypic Synonyms**   - *Argemone barclayana* Penny ex Loudon - *Argemone intermedia* Sweet - *Argemone intermedia* var. *stenopetala* (Rose) Prain - *Argemone intermedia* var. *typica* Prain - *Argemone mexicana* var. *alba* DC. ex Brandegee - *Argemone ochroleuca* var. *stenopetala* (Rose) Shinners - *Argemone ochroleuca* subsp. *stenopetala* (Rose) Ownbey - *Argemone stenopetala* Rose - *Argemone sulphurea* Sweet ex G.Don - *Papaver mexicanum* var. *barclayana* (Penny ex Loudon) Fr. |
| ***Trigonella hamosa*** | **Homotypic Synonyms**   - *Trigonella hamosa* subsp. *uncata* (Boiss. & Noë) C.C. Towns. - *Trigonella uncata* Boiss. & Noë   **Heterotypic Synonyms**   - *Telis uncata* Kuntze |
| ***Galium odoratum*** | **Homotypic Synonyms**   - *Asperula odora* Salisb. - *Asperula odorata* L. - *Chlorostemma odoratum* (L.) Fourr. - *Galium matrisylva* F.H. Wigg.   **Heterotypic Synonyms**   - *Asperula eugeniae* K. Richt. - *Asperula matrisylva* Gilib. - *Asperula odorata* var. *trifida* Sennen - *Asterophyllum asperula* K.F. Schimp. & Spenn. - *Asterophyllum sylvaticum* K.F. Schimp. & Spenn. - *Galium odoratum* var. *eugeniae* (K. Richt.) Ehrend. |
| ***Erucaria hispanica*** | **Homotypic Synonyms**   - *Raphanus hispanicus* (L.) Crantz - *Sinapis hispanica* L.   **Heterotypic Synonyms**   - *Bunias myagroides* L. - *Cakile myagroides* (L.) Poir. - *Cordylocarpus laevigatus* Willd. - *Cordylocarpus tenuifolius* Sm. - *Crucifera aleppica* (Gaertn.) E.H.L. Krause - *Didesmus myagroides* Desv. - *Didesmus pinnatus* DC. - *Erucaria aleppica* Gaertn. - *Erucaria aleppica* var. *latifolia* (DC.) Boiss. - *Erucaria aleppica* var. *polysperma* Boiss. - *Erucaria aleppica* var. *puberula* Boiss. - *Erucaria grandiflora* Boiss. - *Erucaria latifolia* DC. - *Erucaria lineariloba* Boiss. - *Erucaria myagroides* (L.) Halácsy - *Erucaria tenuifolia* (Sm.) DC. |
